# Loss of fragile *WWOX* gene leads to senescence escape and genome instability

**DOI:** 10.1007/s00018-023-04950-1

**Published:** 2023-10-28

**Authors:** Hui-Ching Cheng, Po-Hsien Huang, Feng-Jie Lai, Ming-Shiou Jan, Yi-Lin Chen, Szu-Ying Chen, Wan-Li Chen, Chao-Kai Hsu, Wenya Huang, Li-Jin Hsu

**Affiliations:** 1 Institute of Basic Medical Sciences, College of Medicine, National Cheng Kung University, Tainan, 70101 Taiwan; 2 Department of Biochemistry and Molecular Biology, College of Medicine, National Cheng Kung University, Tainan, 70101 Taiwan; 3 Department of Dermatology, Chi Mei Medical Center, Tainan, 71004 Taiwan; 4 Center for General Education, Southern Taiwan University of Science and Technology, Tainan, 71005 Taiwan; 5 Institute of Biochemistry, Microbiology and Immunology, Chung Shan Medical University, Taichung, 40201 Taiwan; 6 Department of Medical Laboratory Science and Biotechnology, College of Medicine, National Cheng Kung University, Tainan, 70101 Taiwan; 7 Molecular Diagnosis Laboratory, Department of Pathology, National Cheng Kung University Hospital, Tainan, 704302 Taiwan; 8 Department of Dermatology, College of Medicine, National Cheng Kung University, Tainan, 70101 Taiwan; 9 Center of Infectious Disease and Signaling Research, College of Medicine, National Cheng Kung University, Tainan, 70101 Taiwan; 10 Research Center for Medical Laboratory Biotechnology, College of Medicine, National Cheng Kung University, Tainan, 70101 Taiwan

**Keywords:** Tumor suppressor, Replicative arrest, Mononucleotide repeat markers, CDK inhibitors, Promoter hypermethylation, Redox homeostasis

## Abstract

Induction of DNA damage response (DDR) to ensure accurate duplication of genetic information is crucial for maintaining genome integrity during DNA replication. Cellular senescence is a DDR mechanism that prevents the proliferation of cells with damaged DNA to avoid mitotic anomalies and inheritance of the damage over cell generations. Human *WWOX* gene resides within a common fragile site *FRA16D* that is preferentially prone to form breaks on metaphase chromosome upon replication stress. We report here that primary *Wwox* knockout (*Wwox*^−/−^) mouse embryonic fibroblasts (MEFs) and WWOX-knockdown human dermal fibroblasts failed to undergo replication-induced cellular senescence after multiple passages in vitro. Strikingly, by greater than 20 passages, accelerated cell cycle progression and increased apoptosis occurred in these late-passage *Wwox*^−/−^ MEFs. These cells exhibited γH2AX upregulation and microsatellite instability, indicating massive accumulation of nuclear DNA lesions. Ultraviolet radiation-induced premature senescence was also blocked by WWOX knockdown in human HEK293T cells. Mechanistically, overproduction of cytosolic reactive oxygen species caused *p16*^*Ink4a*^ promoter hypermethylation, aberrant p53/p21^Cip1/Waf1^ signaling axis and accelerated p27^Kip1^ protein degradation, thereby leading to the failure of senescence induction in *Wwox*-deficient cells after serial passage in culture. We determined that significantly reduced protein stability or loss-of-function A135P/V213G mutations in the DNA-binding domain of p53 caused defective induction of *p21*^*Cip1/Waf1*^ in late-passage *Wwox*^−/−^ MEFs. Treatment of *N*-acetyl-l-cysteine prevented downregulation of cyclin-dependent kinase inhibitors and induced senescence in *Wwox*^−/−^ MEFs. Our findings support an important role for fragile *WWOX* gene in inducing cellular senescence for maintaining genome integrity during DDR through alleviating oxidative stress.

**Supplementary Information:**

The online version contains supplementary material available at 10.1007/s00018-023-04950-1.

## Introduction

The maintenance of genome integrity is crucial for normal cell physiology and organism survival. Upon DNA lesion generation, the induction of DNA damage response (DDR) for inhibiting cell cycle progression and promoting DNA repair safeguards the genome integrity from the generation of potentially deleterious mutations [1]. The DDR apparatus consists of complicated and sophisticated networks that are required for preserving genetic information and ensuring accurate transmission of DNA through cell generations during replication. If the DNA lesions are beyond repair capacity, the DDR machinery may trigger apoptosis or cellular senescence [2–4].

Cellular senescence is a stress response that has been linked with stimulation of genotoxic agents, telomere shortening or dysfunction, ageing, oxidative stress, oncogene activation, and replication stress [3–6]. This phenomenon was first characterized by a durable arrest of the cell cycle in human fibroblasts serially passaged in culture [7]. The presence of senescent cells has been inferred from a number of senescence markers, such as persistent growth arrest, enlarged and flattened cell morphology, enhanced senescence-associated β-galactosidase (SA-β-gal) activity, and increased expression of cyclin-dependent kinase (CDK) inhibitors p16^Ink4a^ and p21^Cip1/Waf1^ [2, 3, 8].

The p16^Ink4a^ and p53-p21^Cip1/Waf1^ signaling cascades are two central activating pathways of cellular senescence [6, 9–11]. As a barrier to cell proliferation, p16^Ink4a^ blocks cyclin D·CDK4/6 complex-mediated inactivation of retinoblastoma (RB) family proteins to suppress cell cycle progression. The expression of p16^Ink4a^ protein is known to increase with ageing in multiple human tissues, and its inactivation by increased CpG island methylation of the *p16*^*Ink4a*^ promoter region is associated with the escape from senescence induction in human epithelial cells [9, 12, 13]. Upon sensing double-stranded DNA breaks, the DDR stimulates a signaling cascade centered around the damage sensor ataxia telangiectasia-mutated (ATM) kinase that activates p53 to elicit cell cycle arrest and to trigger senescence [1, 11]. After activation, translocation of p53 into the nucleus for the induction of its transcriptional target *p21*^*Cip1/Waf1*^ is pivotal for the establishment of senescence [11]. In turn, p21^Cip1/Waf1^ inhibits CDK2-mediated inactivation of RB, thereby preventing the entry of cells into S phase of the cell cycle. Many DNA-damaging stimuli, such as irradiation, chemotherapeutic drugs, or reactive oxygen species (ROS), engage the ATM-p53-p21^Cip1/Waf1^ axis [14]. Ataxia telangiectasia and Rad3-related protein (ATR) kinase sensing single-stranded DNA breaks during DNA repair or replication stress also activates p53 [15]. Depending on the stress or cell type, the p16^Ink4a^ and p53-p21^Cip1/Waf1^ signaling cascades can act either alone or in combination with each other to induce cellular senescence [2].

Common fragile sites are large chromosomal regions that tend to form gaps or breaks during DNA replication. Alterations at chromosomal fragile sites have been implicated as being causative for human cancers [16, 17]. Interestingly, some genes residing within the chromosomal fragile region encode proteins that are involved in the protection of genome integrity for maintaining cell homeostasis [18–22]. Human *WWOX* gene is located at the common fragile site *FRA16D* and encodes WW domain-containing oxidoreductase [23–25]. Previous studies have shown that WWOX increases the kinase activities of ATM and ATR for facilitating DNA repair [26–28]. WWOX also interacts with Brca1 to support non-homologous end-joining repair of DNA double-strand breaks [29]. Of note, all whole-body homozygous *Wwox* knockout (*Wwox*^−/−^) mice died by four weeks postnatally [30–32]. The cause leading to high embryonic or postnatal lethality in *Wwox*^−/−^ mice is unknown. Early lethal microcephaly syndrome with epilepsy, cerebellar ataxia, mental retardation and profound developmental delay have been observed in pediatric patients with autosomal recessive mutation of *WWOX* [33–38]. WWOX deficiency is also associated with human malignancies and neurodegeneration [39, 40]. However, the molecular pathogenesis that results in the abnormalities in cell homeostasis due to loss of fragile *WWOX* remains largely unclear. In this study, we show that targeted disruption of *Wwox* gene in mouse embryonic fibroblasts (MEFs) facilitates their escape from induction of replicative senescence via inactivating both p16^Ink4a^ and p21^Cip1/Waf1^ and leads to loss of genome integrity after serial passages in cell culture. Treatment of cells with *N*-acetyl-l-cysteine (NAC) prevents aberrant cell cycle progression and triggers senescence in *Wwox*^−/−^ MEFs, indicating that oxidative stress confers the senescence escape in cells null for *Wwox* alleles.

## Materials and methods

### Isolation and culture of MEFs

Whole-body heterozygous *Wwox* exon 1-targeting knockout (*Wwox*^+/−^) mice were generated and interbred as previously described [31]. MEFs isolated from ~ E14.5 fetuses were cultured at 37 °C in 5% CO_2_ in Dulbecco’s modified Eagle’s medium (DMEM; Invitrogen, Carlsbad, CA, USA) supplemented with 10% fetal bovine serum (FBS; Thermo Fisher Scientific, Waltham, MA, USA). The procedures for animal experiments were carried out in strict accordance with an approved protocol from the Institutional Animal Care and Use Committee of National Cheng Kung University, Taiwan.

### Cell line, lentivirus, reagents and plasmids

HEK293T cells obtained from American Type Culture Collection were maintained in DMEM containing 5% FBS at 37 °C. Human dermal fibroblasts were purchased from Lonza (Basel, Switzerland). Lentivirus-mediated gene knockdown was performed as described previously [41] using the sequences listed in Supplementary Table 1. 5-Azacytidine (5-Aza) was obtained from MedChemExpress (#HY-10586; Monmouth Junction, NJ, USA). MG132 was purchased from Calbiochem (#474790; Merck & Co., Inc., Rahway, NJ, USA). NAC and chloroquine diphosphate salt were from Sigma-Aldrich (#A8199 and #C6628, respectively; Burlington, MA, USA). Bleomycin, an inducer of DNA strand breaks, was obtained from Nippon Kayaku Co., Tokyo, Japan. The Cell Counting Kit-8 supplied by Dojindo Molecular Technologies, Inc. (Rockville, MD, USA), and hemocytometer were used to determine the numbers of viable cells in cell proliferation assay.

Human p21^Cip1/Waf1^ and p53 expression vectors were obtained from Addgene (#20814 and #11770, respectively; Watertown, MA, USA). Full-length murine *p16*^*Ink4a*^ was amplified by polymerase chain reaction (PCR) using the complementary DNA (cDNA) template generated from early-passage wild-type MEFs and the primer set listed in Supplementary Table 1, and cloned into the pcDNA3.1 vector digested with BamHI and EcoRI. To generate the plasmid constructs expressing mutant p53, site-directed mutagenesis was performed using Agilent’s QuikChange Lightning kits (Santa Clara, CA, USA) according to the manufacturer’s instructions and the mutation-containing primer sequences (Supplementary Table 1). All expression constructs were confirmed by DNA sequencing. The pGL3 luciferase reporter plasmid containing the 564-bp *p16*^*Ink4a*^ promoter sequence (− 869 to − 305) was a generous gift from Dr. Sumit K. Chanda at the Sanford Burnham Prebys Medical Discovery Institute [42]. The WWP-Luc (*p21*^*Cip1/Waf1*^ promoter) construct was obtained from Dr. Bert Vogelstein (#16451; Addgene, Watertown, MA, USA) [43]. The pp53-TA-Luc luciferase-based reporter plasmid harboring a p53 response element (#PT3511-5W; Clontech, Mountain View, CA, USA) was used for monitoring p53-mediated signaling in MEFs. For plasmid DNA transfection, cells were electroporated with the above-mentioned construct or a control vector using a BioRad Gene Pulser System (Hercules, CA, USA) as described previously [44], and cultured at 37 °C in 96-well plates or 10-cm dishes.

### Flow cytometric analysis

For bromodeoxyuridine (BrdU) labeling, 8 × 10^5^ MEFs in 3 ml were cultured overnight in 6-cm dishes, pulsed by the addition of 1 mM BrdU solution (10 μl; BD Pharmingen, Franklin Lakes, NJ, USA) and further incubated at 37 °C for 2 h. Cells were trypsinized and centrifuged at 2000 rpm for 7 min at 4 °C and the supernatants were discarded. After PBS wash, cells were resuspended in 100 μl of BD Cytofix/Cytoperm buffer, and incubated at 4 °C for 12 h. After wash with BD Perm/Wash buffer, cells were treated with 20 U/ml DNase I at 37 °C for 1 h. The cells were washed with BD Perm/Wash buffer and then stained with FITC-conjugated anti-BrdU (10 μl antibody diluted to 50 μl using BD Perm/Wash buffer; BD Pharmingen) for 30 min at room temperature in the dark. For propidium iodide (PI) staining, cells were trypsinized, washed with PBS, fixed with 70% ethanol/PBS for 30 min at 4 °C and centrifuged at 2000 rpm for 10 min at room temperature. After removal of the supernatant, cells were stained with 500 μl of the staining solution containing 40 μg/ml PI (Sigma-Aldrich) and 100 μg/ml RNase A (Roche, Basel, Switzerland) in PBS for 20 min at room temperature in the dark. For dihydroethidium (DHE) staining, the trypsinized MEFs were washed with PBS and stained with 500 μl of DHE staining solution (5 μM in PBS; Invitrogen, Carlsbad, CA, USA) for 20 min at room temperature in the dark. Cells were analyzed using a FACS Calibur (Becton Dickinson, Mountain View, CA, USA) with excitation set at 488 nm.

### Immunofluorescence, annexin V staining and micronucleus assay

MEFs on glass coverslips were fixed with 3.7% formaldehyde/PBS at room temperature for 15 min, washed twice with PBS, and incubated with a permeabilization buffer (0.1% saponin, 1% bovine serum albumin and 0.1% NaN_3_ in PBS) at 4 °C for 1 h. Cells were stained with anti-γH2AX antibody (#9718, 1:200 dilution in permeabilization buffer; Cell Signaling Technology, Danvers, MA, USA) at 4 °C overnight. After PBS wash, cells were further stained with an Alexa Fluor 594-conjugated donkey anti-rabbit IgG antibody (#A21207, 1:2000 dilution; Invitrogen, Carlsbad, CA, USA) in the dark at room temperature for 2 h. The samples incubated only with secondary antibody were used as negative controls. After PBS wash, the nuclei were stained with 4′,6-diamidino-2-phenylindole (DAPI; 1 μg/ml in PBS, Sigma-Aldrich) at room temperature for 5 min. After wash, the samples were mounted on a glass slide and the γH2AX-positive cells were visualized under an Olympus fluorescence microscope (FV1000, Tokyo, Japan). For annexin V-binding assay, MEFs cultured in 12-well plates were washed twice with cold PBS and incubated with phycoerythrin-conjugated annexin V (BD #559763) diluted in annexin V-binding buffer for 20 min at room temperature in the dark. The exposure of cell surface phosphatidylserine was examined by fluorescence microscopy. For micronucleus assay, MEFs on glass coverslips were treated with 200 nM bleomycin at 37 °C for 48 h to induce DNA strand breaks. After incubation, cells were fixed with 3.7% formaldehyde/PBS for 15 min at room temperature, washed with PBS and stained with 1 μg/ml DAPI in PBS. The presence of micronuclei was examined by fluorescence microscopy (Olympus, Tokyo, Japan).

### Hypoxanthine phosphoribosyltransferase (*Hprt*) mutation analysis and microsatellite instability assay

Genomic DNA isolated from MEFs using a Quick-DNA Miniprep kit (#D3024; Zymo Research, Irvine, CA, USA) was utilized for PCR detection of Y-chromosomal genes *Ube1, Sry* and *Zfy*. Sequences of the primer sets used are shown in Supplementary Table 1. To analyze the mutation rate in X-linked *Hprt* gene, the MEFs derived from male mouse embryo were seeded at 1.5 × 10^6^ cells/dish in 10-cm dishes and cultured with the presence of 5 μg/ml 6-thioguanine (#15774; Cayman, Ann Arbor, MI, USA) at 37 °C for 14 days with medium changed every 3 to 4 days. Cells were stained with 1% crystal violet in 20% methanol for 1 h at room temperature, and the culture dishes were rinsed with water to remove the unbound dye and detached cells. The cell-bound crystal violet was dissolved with 2 ml of acetic acid solution and the eluate (100 μl) was analyzed by the measurement of optical density at 570 nm using a microplate reader (Synergy HT, BioTek, Winooski, VT, USA).

Microsatellite instability in MEFs was tested with five previously described mononucleotide repeats *Bat26*, *Bat30*, *Bat37*, *Bat64*, and *Bat67* [45, 46]. PCR amplification of the mononucleotide repeats was carried out using MEF genomic DNA (30 ng), a DNA polymerase (Super Therm Gold Master Mix; Bionovas Biotechnology, Toronto, ON, Canada) and the primer sets listed in Supplementary Table 1. The amplified fragments were separated by capillary electrophoresis using a QIAxcel DNA screening kit (Qiagen Life Sciences, Hilden, Germany).

### SA-β-gal staining

The SA-β-gal staining kit (#9860; Cell Signaling Technology, Danvers, MA, USA) was utilized to detect senescent cells according to the manufacturer’s protocol. In brief, MEFs were seeded and cultured overnight in 24-well plates. Cells were washed twice with cold PBS and fixed with the fixative solution for 15 min at room temperature. After PBS wash, cells were incubated with a staining solution (pH 5.9–6.1) containing 1 mg/ml X-gal and the supplements A and B in an oven set at 37 °C for two days. The blue senescent cells were visualized under a bright-field microscope (Olympus, Tokyo, Japan).

### RNA extraction, reverse transcription PCR, real-time PCR and gene expression profiling

RNA samples were extracted using TRIZOL reagent (Invitrogen, Carlsbad, CA, USA) according to the manufacturer’s instruction. Reverse transcription PCR and quantitative real-time PCR (qPCR) were performed as described previously [44], using the primer pairs listed in Supplementary Table 1.

For microarray analysis, an Agilent Low Input Quick Amp Labeling kit was utilized to amplify the total RNA (0.2 μg) isolated from MEFs of passage one and label with Cy3. The Cy3-labeled cRNA was fragmented to an average length of 50–100 nucleotides and then hybridized to the Agilent SurePrint G3 Mouse GE 8 × 60K microarray at 65 °C for 17 h. After washing and scanning at 535 nm for Cy3, the microarray images were analyzed using a Feature Extraction 10.5.1.1 software (Agilent Technologies, Santa Clara, CA, USA).

### Co-immunoprecipitation, subcellular fractionation and western blot analysis

Equal amount of whole cell lysates or specific antibody-precipitated cellular protein complexes were prepared as described previously [44]. A ProteoExtract^®^ Subcellular Proteome Extraction kit (Calbiochem #539790; Merck & Co., Inc., Rahway, NJ, USA) was used to extract subcellular protein samples according to the manufacturer’s protocol. Standard SDS-PAGE and subsequent western blotting procedures were carried out to resolve proteins using the following antibodies: anti-caspase 3 (#9662, 1:1000; Cell Signaling Technology, Danvers, MA, USA), anti-p16^Ink4a^ (#sc-1207, 1:500; Santa Cruz, Dallas, TX, USA), anti-p19^Arf^ (#20080, 1:1000; GeneTex, Irvine, CA, USA), anti-p21^Cip1/Waf1^ (#sc-471, 1:500; Santa Cruz), anti-p27^Kip1^ (#2552, 1:1000; Cell Signaling Technology), anti-phospho-p27^Kip1^ (Thr187; #37-9700, 1:500; Zymed Laboratories, San Francisco, CA, USA), anti-p53 (#2524, 1:1000; Cell Signaling Technology), anti-phospho-p53 (human Ser15/murine Ser18; #21431, 1:1000; GeneTex), anti-MDM2 (#100531, 1:1000; GeneTex), anti-green fluorescence protein (GFP) (GeneTex #21218 for immunoprecipitation, and Santa Cruz #sc-9996 for immunoblotting, 1:1000), anti-histone H3 (#9715, 1:1000; Cell Signaling Technology), anti-GAPDH (#100118, 1:5000; GeneTex), and anti-β-actin (#A5441, 1:10,000; Sigma-Aldrich). Our generated rabbit polyclonal antibodies against a synthetic peptide corresponding to amino acids 89–107 of murine WWOX were also used for immunoblotting [39, 44]. Horseradish peroxidase-conjugated goat anti-rabbit IgG or horse anti-mouse IgG (#7074 or #7076, respectively; 1:5000 dilution; Cell Signaling Technology) were used as secondary antibodies. The detection of immunoblots was performed using an enhanced chemiluminescence kit (Millipore, Burlington, MA, USA) as described previously [44]. Quantitative densitometry of immunoblots was analyzed using the ImageJ software (National Institutes of Health, Bethesda, MD, USA).

### Luciferase reporter assay

A control thymidine kinase promoter-driven renilla luciferase reporter plasmid (pRL-TK) and a firefly luciferase reporter plasmid containing a human *p16*^*Ink4a*^ or *p21*^*Cip1/Waf1*^ promoter sequence or a p53 response element were transfected into MEFs by electroporation. The cells were incubated at 37 °C for 18 h, and the induction of luciferase activities was measured using a dual-luciferase assay kit (Promega, Madison, WI, USA) according to the manufacturer’s protocol.

### DNA methylation analysis

To quantify the CpG methylation status within *p16*^*Ink4a*^ and *p21*^*Cip1/Waf1*^ promoters in MEFs, bisulfite conversion of genomic DNA was performed using an EZ DNA methylation kit (Zymo Research) as described previously [47]. PCR amplification of the targeted promoter regions was performed using the primers listed in Supplementary Table 1. After in vitro transcription using the PCR products as the template and T7 RNA polymerase, uracil-specific cleavage of the RNA transcripts by RNase A was conducted and the sizes of the resulting fragments were determined by MALDI-TOF mass spectrometry (MassARRAY Analyzer, Agena Bioscience, San Diego, CA, USA). The data were analyzed using an EpiTYPER software.

### Chromatin immunoprecipitation (ChIP)

Cells in 15-cm dishes were fixed with 1% formaldehyde/PBS for 15 min at room temperature and collected for centrifugation at 3000 rpm for 2 min at 4 °C. The cell pellets were mixed with a lysis buffer (4 ml) containing 50 mM Tris (pH 8.0), 2 mM EDTA, 0.1% NP40, 10% glycerol and protease inhibitors using a syringe with 26G needle. After centrifugation at 13,000 rpm for 1 min at 4 °C, the pellets were suspended in a buffer (2 ml) containing 50 mM Tris (pH 8.0), 1.6 mM EDTA, 150 mM NaCl, 0.38% NP40, 0.25% SDS and protease inhibitors. By sonication and centrifugation, chromatin fragments in the supernatant with an average size of 500–1000 bp were prepared. Following determination of the protein concentration, an equal amount of the samples (containing 500 μg proteins) was mixed with anti-p53 (1 μl; #2524; Cell Signaling Technology) or a control IgG and the samples were rotated at 4 °C overnight. The mixtures were further incubated with protein A/G beads at 4 °C for 2 h, and the beads were washed with a high salt buffer (20 mM Tris, 2 mM EDTA, 0.5% NP40, 0.1% SDS, 0.5 M NaCl, pH 8.0) once and a low salt buffer (10 mM Tris, 1 mM EDTA, pH 8.0) for three times. The precipitates were incubated with a buffer (500 μl) containing 10 mM Tris, 0.1 mM EDTA and 1% SDS (pH 8.0) at 65 °C overnight, followed by the treatment of proteinase K (100 μg/sample; Abcam, Cambridge, UK) at 50 °C for 1 h. The captured genomic DNA was extracted using phenol/chloroform/isoamyl alcohol and precipitated by ethanol according to the standard protocols. The DNA samples were dissolved in 40 μl TE buffer and subjected to PCR using the primers listed in Supplementary Table 1.

### Statistical analysis

Data were presented as the means ± standard deviation (SD). Statistical significance was determined using one-way or two-way ANOVA, one or two-tailed paired *t* test, or two-tailed *t* test as described in figure legends. The differences between groups were considered significant when the *P* values were less than 0.05.

## Results

### MEFs null for *Wwox* gene exhibit increased genome instability and aberrant cell growth and apoptosis after serial passages in culture

**Fig. 1:**
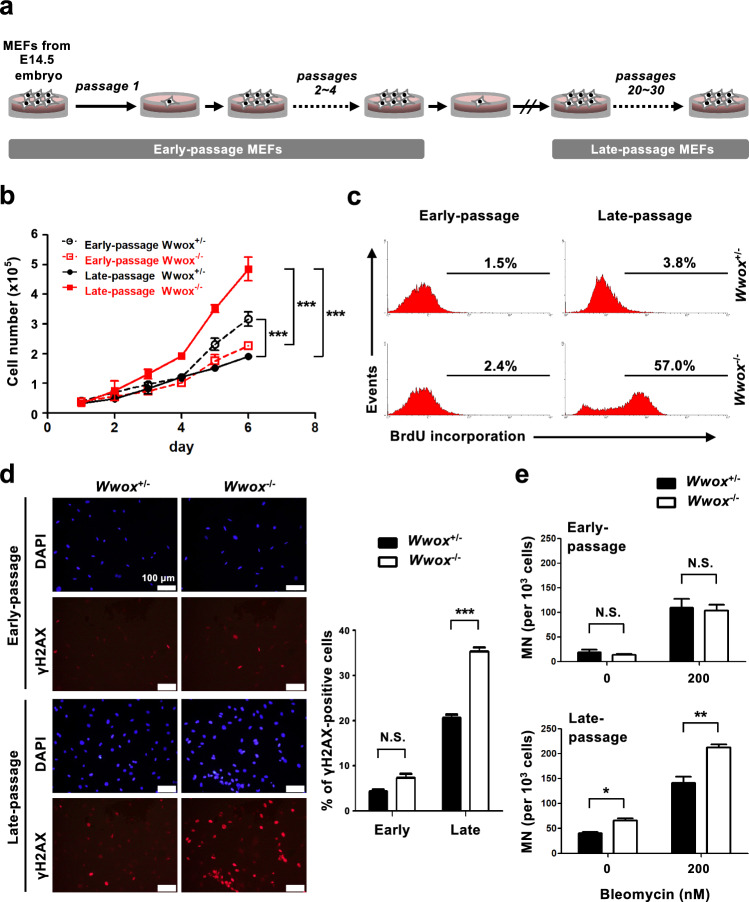
MEFs null for *Wwox* gene exhibit increased genome instability and aberrant cell growth after serial passages in culture. **a** The experimental setup for serial passage culture of MEFs. **b** Cell growth rates of *Wwox*^+/−^ and *Wwox*^−/−^ MEFs at early- and late-passages. ***P ≤ 0.005; Two-way ANOVA. **c** *Wwox*^+/−^ and *Wwox*^−/−^ MEFs at early- and late-passages were cultured in a low serum condition for 72 h. After serum starvation, MEFs were treated with 10% FBS for 12 h and pulsed with 10 μM BrdU during the final 2 h of culture. MEFs were fixed, permeabilized, stained with a FITC-conjugated anti-BrdU antibody and analyzed using a FACS Calibur. The percentages of BrdU-positive MEFs were quantified using the WinMDI software. **d** Immunofluorescent staining of γH2AX (red) in *Wwox*^+/−^ and *Wwox*^−/−^ MEFs at early- and late-passages. The nuclei were stained with DAPI (blue). Representative images obtained from at least three independent experiments are shown. The quantitative results of γH2AX-positive cells are shown in the right panel. Scale bars = 100 µm. *N.S.* not significant; ***P ≤ 0.005; One-way ANOVA. **e** *Wwox*^+/−^ and *Wwox*^−/−^ MEFs at early- and late-passages were treated with the indicated doses of bleomycin, and micronucleus (MN) analysis was performed. *N.S.* not significant; *P ≤ 0.05; **P ≤ 0.01; Two-tailed *t* test

MEFs freshly isolated from control or *Wwox* knockout mouse embryos at E14.5 were cultured on dishes and one sixth of the MEFs were passed every 5 days before the cells reached confluent. The cells at passages 1–4 were defined as early-passage MEFs and passages 20–30 with an average of 25 as late-passage MEFs (Fig. 1a). The control MEFs expressing Wwox protein gradually lost their replicative potential after serial passages in cell culture [48–50], whereas rapid and robust cell proliferation was observed in the late-passage *Wwox*^−/−^ MEFs (Fig. 1b). Cell cycle analysis revealed a higher S-phase fraction in late-passage *Wwox*^−/−^ MEFs compared to the control MEFs (33.1% knockout vs. 14.6% control; Supplementary Fig. 1a). To test whether long-term passage culture of MEFs in *Wwox*-null status leads to accelerated cell cycle entry, MEFs were synchronized in G0/G1 phase by serum deprivation for 72 h and then stimulated by the addition of serum growth factors. Compared with late-passage control MEFs, we determined increased percentages of cells progressing into S and G2/M phases of the cell cycle in late-passage *Wwox*^−/−^ MEFs after treatment with serum for 12 and 18 h (Supplementary Fig. 1a). Likewise, a markedly increased population of actively proliferating cells in serum-stimulated late-passage *Wwox*^−/−^ MEFs was measured by a high extent of BrdU incorporation into the newly synthesized DNA (Fig. 1c). No significant differences were detected between the early-passage *Wwox*^−/−^ and control MEFs (Fig. 1c, Supplementary Fig. 1a). Intriguingly, we also detected higher amounts of apoptotic cells in late-passage *Wwox*^−/−^ MEFs than the control cells, as evidenced by the increased proportion of cells in sub G0 population upon cell cycle analysis, binding of annexin V to cell surface phosphatidylserine, and caspase-3 cleavage (Supplementary Fig. 1a–c).

Since some genetically unstable MEFs have both an increased rate of proliferation and a higher level of apoptosis [51], we analyzed whether late-passage *Wwox*^−/−^ MEFs exhibit the phenotype of genome instability. γH2AX, the phosphorylated form of histone H2AX, is required for the assembly of repair proteins at DNA lesion sites during DDR and is commonly used as a sensitive marker for DNA double-strain breaks and genome instability in the cells [52, 53]. By immunofluorescent staining, we determined that γH2AX was highly expressed in late-passage *Wwox*^−/−^ MEFs (Fig. 1d). Moreover, compared with the control cells, an increased extent of bleomycin-induced micronucleus formation, an indicator of chromosomal damages, was detected in late-passage *Wwox*^−/−^ MEFs (Fig. 1e). To further examine the mutation frequencies in MEFs, 6-thioguanine resistance acquired by X-linked *Hprt* gene mutations was used in this study as a global mutation indicator [54]. PCR detection of murine Y-chromosomal genes *Ube1*, *Sry* and *Zfy* using the primers listed in Supplementary Table 1 was performed for gender typing. After 6-thioguanine treatment, the significantly increased numbers of viable cells in the late-passage *Wwox*^−/−^ MEF cultures from male mouse embryo revealed a high incidence of *Hprt* mutations (Supplementary Fig. 2a–c). Replication of microsatellite DNA sequences is error-prone because of DNA polymerase slippage that may create mutations resulting from single base mismatches or short insertions and deletions [45, 46]. We determined accumulation of length variations in the microsatellite repetitive DNA sequences in late-passage *Wwox*^−/−^ MEFs using a panel of mononucleotide repeat markers for detecting the phenotype of microsatellite instability (Supplementary Fig. 2d). Together, our results suggest that genome instability in *Wwox*-null MEFs upon serial passages in culture may lead to accumulation of deleterious DNA lesions and aberrations in cell cycle progression and apoptosis.

### *Wwox*-deficient cells fail to enter cellular senescence

**Fig. 2:**
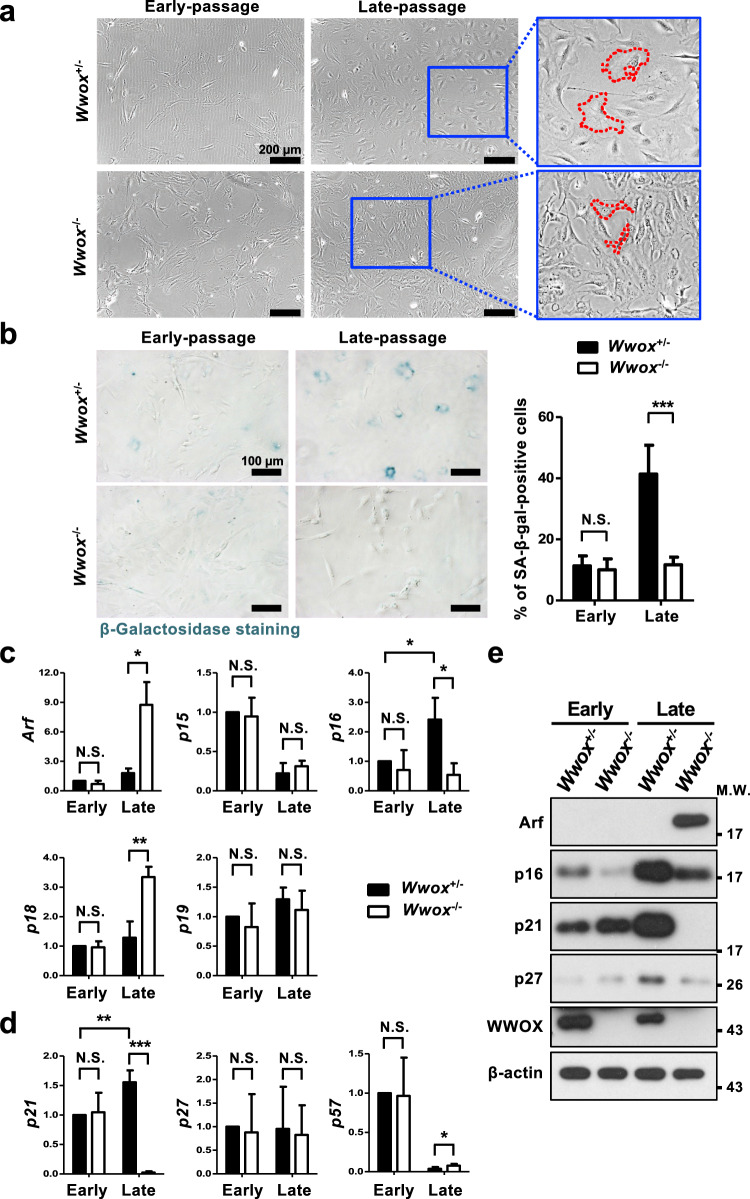
*Wwox*-deficient MEFs fail to enter cellular senescence. **a** Cellular morphology of *Wwox*^+/−^ and *Wwox*^−/−^ MEFs at early- and late-passages. Scale bars = 200 µm. **b** SA-β-gal staining of *Wwox*^+/−^ and *Wwox*^−/−^ MEFs at early- and late-passages. The percentages of SA-β-gal-positive senescent MEFs are shown in the right panel. Scale bars = 100 µm. *N.S.* not significant; ***P ≤ 0.005; One-way ANOVA. **c**, **d** The relative mRNA expression levels of INK (**c**) and KIP/CIP (**d**) CDK family genes in MEFs were determined by quantitative real-time PCR. *N.S.* not significant; *P ≤ 0.05; **P ≤ 0.01; ***P ≤ 0.005; Two-tailed *t* test. **e** The protein expression levels of p19^Arf^, p16^Ink4a^, p21^Cip1/Waf1^, p27^Kip1^ and WWOX in *Wwox*^+/−^ and *Wwox*^−/−^ MEFs at early- and late-passages were determined by western blotting. β-actin was used as an internal control

Normal human primary cells undergo a limited number of divisions in culture and enter a quiescent but viable state called replicative senescence [7]. Replicative arrest or slowed growth after a limited period of proliferation in primary MEF culture due to DNA damage accumulation has become an experimental model system for finding the molecular cues underlying cellular senescence [50, 55–58]. After 20 passages in culture, the control MEFs displayed moderately increased DDR marker γH2AX (Fig. 1d), and lost their original fibroblast morphology by acquiring a flattened and enlarged feature (Fig. 2a). In stark contrast, the late-passage MEFs null for *Wwox* still exhibited a spindle-shaped appearance (Fig. 2a). Increased SA-β-gal activity was detected in the control, but not *Wwox*^−/−^ MEFs at late passages (Fig. 2b). We also examined ultraviolet radiation-induced senescence in human HEK293T cells. Compared with the control cells, knockdown of WWOX protein expression significantly blocked the induction of senescence in HEK293T cells (Supplementary Fig. 3). Similar results were observed in WWOX-knockdown human primary dermal fibroblasts (Supplementary Fig. 4a, b).

Moreover, we detected less amounts of *p16*^*Ink4a*^ and *p21*^*Cip1/Waf1*^ mRNA in late-passage *Wwox*^−/−^ MEFs (Fig. 2c, d, Supplementary Fig. 5). However, mRNA levels of *Arf* and CDK inhibitors *p18* in the INK family and *p57* in the KIP/CIP family were increased in late-passage *Wwox*^−/−^ MEFs, indicating that transcriptional induction of these cell cycle checkpoint proteins by DDR remains intact in these cells (Fig. 2c, d, Supplementary Fig. 5). The protein expression levels of p16^Ink4a^, p21^Cip1/Waf1^ and p27^Kip1^ were increased in the senescent control MEFs after the continuous cell culture process, whereas the late-passage *Wwox*^−/−^ MEFs had markedly reduced protein levels of these CDK inhibitors in comparison with the late-passage control cells (Fig. 2e). Significantly decreased protein amounts of CDK inhibitors p16^Ink4a^ and p27^Kip1^ were also observed in WWOX-knockdown human cells (Supplementary Fig. 4c). DDR has been shown to increase p27^Kip1^ protein stability [59, 60]. We determined comparable *p27*^*Kip1*^ mRNA levels among all groups of MEFs (Fig. 2d, Supplementary Fig. 5b). MG132 treatment blocked p27^Kip1^ protein downregulation in late-passage *Wwox*^−/−^ MEFs (Supplementary Fig. 6a), suggesting that p27^Kip1^ protein degradation is accelerated through proteasomal pathway to prevent its accumulation in late-passage *Wwox*^−/−^ MEFs. Increased phosphorylation of p27^Kip1^ at Thr187 was examined in late-passage *Wwox*^−/−^ MEFs (Supplementary Fig. 6b). p27^Kip1^ protein phosphorylation at Thr187 may facilitate its polyubiquitination by E3 ligase SCF^Skp2^ [61, 62].

### Defective signaling for transcriptional activation of *p21*^*Cip1/Waf1*^ and CDK inhibitor promoter methylation prevent senescence induction in late-passage *Wwox*^−/−^ MEFs

**Fig. 3:**
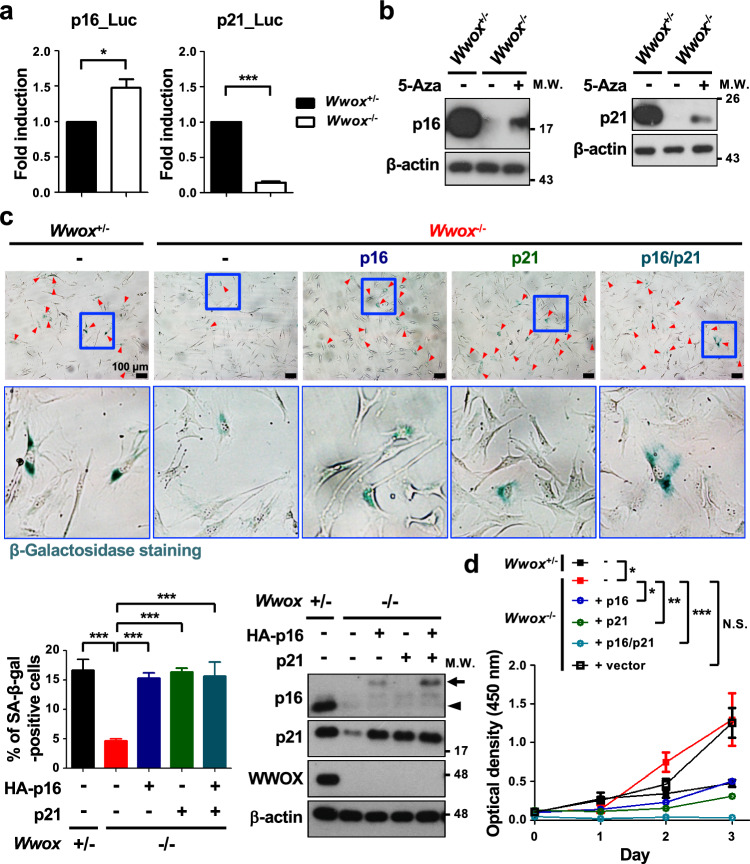
Defective induction of *p16*^*Ink4a*^ and *p21*^*Cip1/Waf1*^ causes senescence escape in late-passage *Wwox*^−/−^ MEFs. **a** The upstream signaling for transcriptional activation of *p21*^*Cip1/Waf1*^ was defective in late-passage *Wwox*^−/−^ MEFs. The *p16*^*Ink4a*^ promoter (in pGL3-Luc) or *p21*^*Cip1/Waf1*^ promoter (in pWWP-Luc) construct was transfected into MEFs. Luciferase activities were determined at 16 h after transfection. Data are presented as mean ± SD from three independent experiments. *P ≤ 0.05; ***P ≤ 0.005; Two-tailed *t* test. **b** MEFs were treated with or without a DNA methylation inhibitor 5-Aza (10 μM) for 24 h. The protein expression levels of p16^Ink4a^ or p21^Cip1/Waf1^ were determined by western blotting. β-actin was used as an internal control. **c** SA-β-gal staining of late-passage *Wwox*^+/−^, *Wwox*^−/−^ and *Wwox*^−/−^ MEFs expressing ectopic HA-tagged mouse p16^Ink4a^ and/or human p21^Cip1/Waf1^. The lower panel pictures are the magnified images from the blue boxed areas in the upper panel pictures. The percentages of SA-β-gal-positive senescent cells (red arrowheads) are shown in the bottom left graph. The protein expression of p16^Ink4a^, p21^Cip1/Waf1^ and WWOX was detected by western blotting. β-actin was used as an internal control. The black arrow and arrowhead indicate ectopic and endogenous p16^Ink4a^, respectively. Scale bars = 100 µm. ***P ≤ 0.005; Two-tailed *t* test. **d** The cell growth of late-passage *Wwox*^−/−^ MEFs was suppressed by ectopic expression of mouse p16^Ink4a^ and/or human p21^Cip1/Waf1^, as determined using the Cell Counting Kit-8. *N.S.* not significant; *P ≤ 0.05; **P ≤ 0.01; ***P ≤ 0.005; Two-tailed *t* test

To further investigate the mechanism of CDK inhibitor downregulation for senescence escape in late-passage *Wwox*^−/−^ MEFs, we examined DDR-triggered signaling that stimulates promoter activation of *p16*^*Ink4a*^ and *p21*^*Cip1/Waf1*^ in the cells. After transfecting a reporter plasmid containing the *p16*^*Ink4a*^ promoter sequence, we detected a higher luciferase activity in late-passage *Wwox*^−/−^ MEFs than the control cells (Fig. 3a), indicating that the signal for stimulating *p16*^*Ink4a*^ gene activation is enhanced in the cells null for *Wwox*. Intriguingly, the reporter activity of *p21*^*Cip1/Waf1*^ promoter showed a robust decrease in late-passage *Wwox*^−/−^ MEFs in a luciferase reporter assay (Fig. 3a), suggesting that the upstream signal for *p21*^*Cip1/Waf1*^ induction is defective in these cells. To study whether the decreased *p16*^*Ink4a*^ and *p21*^*Cip1/Waf1*^ expression in *Wwox*^−/−^ MEFs is due to promoter hypermethylation, we demonstrated that the reduced protein expression of both p16^Ink4a^ and p21^Cip1/Waf1^ in late-passage *Wwox*^−/−^ MEFs was reversed by treating the cells with a DNA methylation inhibitor 5-Aza (Fig. 3b). Ectopic expression of p16^Ink4a^ and/or p21^Cip1/Waf1^ in late-passage *Wwox*^−/−^ MEFs led to increased SA-β-gal activity and suppression of cell growth (Fig. 3c, d), suggesting that the loss of p16^Ink4a^ and p21^Cip1/Waf1^ expression during serial passage in culture is crucial for the senescence escape of *Wwox*^−/−^ MEFs.

### Aberrant p53/p21^Cip1/Waf1^ axis results in decreased senescence induction in *Wwox*^−/−^ MEFs

**Fig. 4:**
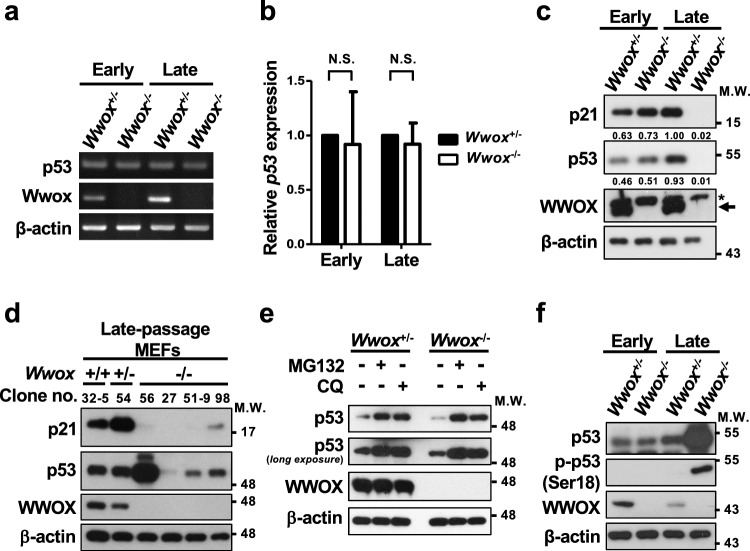
Aberrant p53/p21^Cip1/Waf1^ axis leads to decreased *p21*^*Cip1/Waf1*^ induction in late-passage *Wwox*^−/−^ MEFs. **a**, **b** The mRNA expression levels of p53 in MEFs were determined by reverse transcription PCR (**a**) and quantitative real-time PCR (**b**). Data are presented as mean ± SD from three independent experiments. *N.S.* not significant; One-tailed paired *t* test. **c** The protein expression of p21^Cip1/Waf1^, p53 and WWOX in *Wwox*^+/−^ and *Wwox*^−/−^ MEFs at early- and late-passages was determined by western blotting. The arrow indicates the endogenous WWOX protein (46 kDa) and the asterisk represents a non-specific band arising from the MEF lysates. β-actin was used as a loading control. **d** The protein levels of p21^Cip1/Waf1^, p53 and WWOX in late-passage *Wwox*^+/+^, *Wwox*^+/−^ and various *Wwox*^*−/−*^ MEF clones were examined by western blotting. **e** The late-passage *Wwox*^+/−^ (clone no. 23) and *Wwox*^*−/−*^ (clone no. 53-6) MEFs were treated with 5 μM MG132 or 100 μM chloroquine (CQ) for 14 h. The protein expression of p53, p21^Cip1/Waf1^ and WWOX was determined by western blotting. **f** Protein phosphorylation of murine p53 at Ser18 in *Wwox*^+/−^ (clone no. 54) and *Wwox*^−/−^ (clone no. 56) MEFs at early- and late-passages was determined by western blotting. β-actin was used as an internal control

The induction of *p21*^*Cip1/Waf1*^ gene expression is majorly regulated by transcription factor p53 in response to various stress stimuli [63]. As a reduced signal transduction capacity for inducing *p21*^*Cip1/Waf1*^ promoter activation in late-passage *Wwox*^−/−^ MEFs was detected using a reporter assay (Fig. 3a), we further assessed the expression and function of p53. Our results revealed comparable expression levels of *p53* mRNA between the control and *Wwox*^−/−^ MEFs (Fig. 4a, b). After serial passage in culture, an increase in p53 protein level in late-passage control MEFs was associated with robust expression of p21^Cip1/Waf1^ protein probably due to the induction of DDR signal (Fig. 4c). Downregulation of p53 protein, accompanied by decreased p21^Cip1/Waf1^ expression, was observed in all late-passage *Wwox*^−/−^ MEF clones except the clone no. 56 (Fig. 4d). Treatment of protein degradation inhibitor MG132 or chloroquine reversed protein downregulation of p53 in these late-passage *Wwox*^−/−^ clones (Fig. 4e). Upon DNA damage, phosphorylation of human p53 at Ser15/Ser37 or murine p53 at Ser18 by serine/threonine protein kinases ATM, ATR and DNA-PK causes the dissociation of p53 from its key negative regulator, the E3 ubiquitin ligase MDM2, thereby promoting rapid accumulation and functional activation of p53 [64–66]. Intriguingly, the increased p53 protein expression and phosphorylation at Ser18 in late-passage *Wwox*^−/−^ MEF clone no. 56 failed to induce p21^Cip1/Waf1^ expression (Fig. 4d, f).

**Fig. 5:**
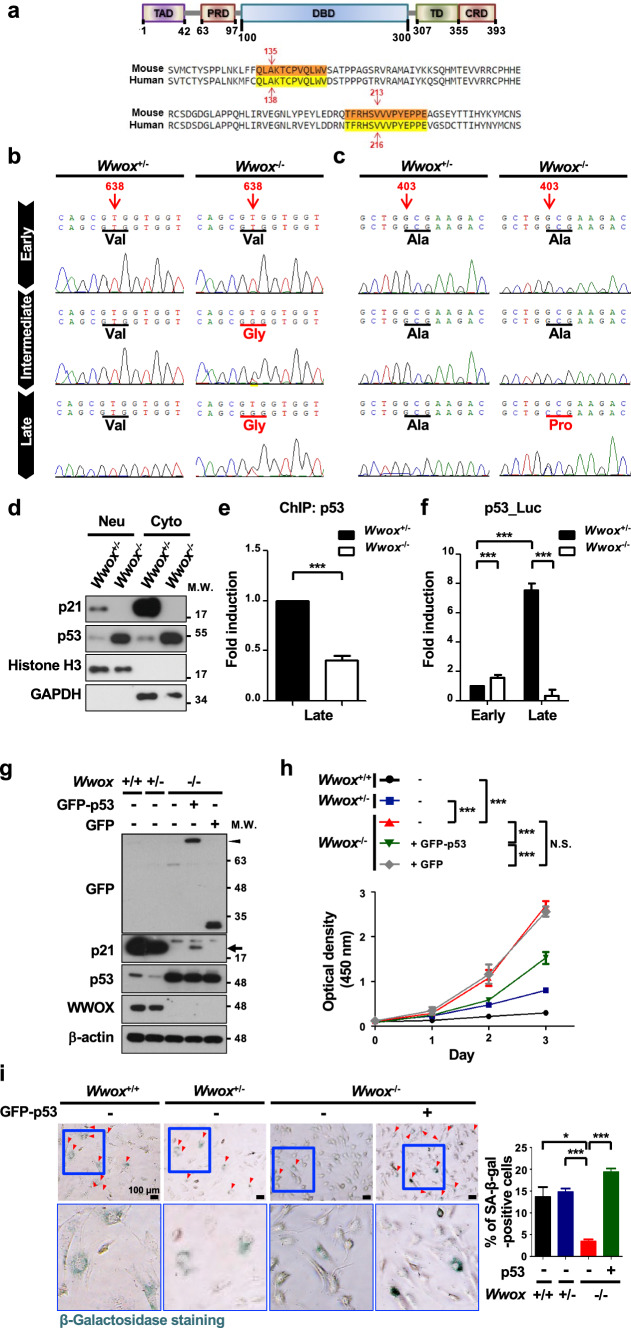
p53 inactivation causes the failure of senescence induction in *Wwox*^−/−^ MEFs. **a** The upper panel is the schematic diagram of p53 protein. The amino acid sequence alignment of human and mouse p53 DBD is shown in the lower panel. TAD, transactivation domain; PRD, proline-rich domain; DBD, DNA-binding domain; TD, tetramerization domain; CRD, C-terminal regulatory domain. **b**, **c** The sequencing results of *p53* gene coding region from *Wwox*^+/−^ (clone no. 54) and *Wwox*^−/−^ (clone no. 56) MEFs at passages 1 (early), 8 (intermediate) and 20 (late) are shown. In **b**, the nucleotide 638 from the translational start site of murine *p53* changed from thymine to guanine, leading to a substitution of valine to glycine at position 213 in the intermediate and late-passage *Wwox*^−/−^ MEF clone no. 56. In **c**, the change of nucleotide 403 from guanine to cytosine resulted in a substitution of alanine to proline in p53 in the late-passage *Wwox*^−/−^ MEF clone no. 56. **d** Subcellular protein fractionation and western blotting were performed to examine the nuclear and cytosolic expression of p53 and p21^Cip1/Waf1^ in late-passage *Wwox*^+/−^ and *Wwox*^−/−^ MEFs (clone no. 54 and 56, respectively). Histone H3 and GAPDH were used as nuclear and cytosolic protein controls, respectively. **e** ChIP assay was conducted using the chromatin isolated from late-passage *Wwox*^+/−^ and *Wwox*^−/−^ MEFs (clone no. 54 and 56, respectively). The immunoprecipitated chromatin using a control or anti-p53 antibody was analyzed for the presence of *p21*^*Cip1/Waf1*^ promoter sequence (from − 200 to 98) by real-time PCR using the specific primers shown in Supplementary Table 1. The quantitative results of ChIP assay were normalized by the input control groups. Data are presented as mean ± SD from three independent experiments. ***P ≤ 0.005; Two-tailed paired *t* test. **f** A reporter construct containing a p53-driven promoter sequence (pp53-TA-Luc) was transfected into MEFs. The thymidine kinase promoter-Renilla luciferase reporter plasmid (pRL-TK) was used as an internal control. Luciferase activities were determined at 16 h after transfection. Data are presented as mean ± SD from three independent experiments. ***P ≤ 0.005; Two-tailed *t* test. **g** The protein expression of endogenous p21^Cip1/Waf1^ (black arrow), p53 and WWOX and ectopic GFP-tagged human p53 (GFP-p53; black arrowhead) in late-passage *Wwox*^+/+^*, Wwox*^+/−^, *Wwox*^−/−^ MEFs (clone no. 32-5, 54 and 56, respectively) was examined by western blotting. β-actin was used as an internal control. M.W., molecular weight. **h** Cell growth rates of late-passage *Wwox*^+/+^*, Wwox*^+/−^, *Wwox*^−/−^ (clone no. 32-5, 54 and 56, respectively) and *Wwox*^−/−^ MEFs expressing ectopic GFP-p53 or GFP protein. *N.S.* not significant; ***P ≤ 0.005; Two-tailed *t* test. **i** SA-β-gal staining of late-passage *Wwox*^+/+^*, Wwox*^+/−^, *Wwox*^−/−^ (clone no. 32-5, 54 and 56, respectively) and *Wwox*^−/−^ MEFs expressing ectopic GFP-p53 protein. The lower panel pictures are the magnified images from the blue boxed areas in the upper panel. The percentages of SA-β-gal-positive senescent cells (red arrowheads) are shown at the right panel. Scale bars = 100 µm. *P ≤ 0.05; ***P ≤ 0.005; Two-tailed *t* test

As *Wwox* knockout led to genome instability in MEFs (Fig. 1d, e), we analyzed the DNA sequences of the entire p53 coding region, and identified two point mutations in the DNA-binding domain (DBD) of murine p53 in the late-passage *Wwox*^−/−^ MEF clone no. 56 (Fig. 5a). The first mutation found at nucleotide 638 in this *Wwox*^−/−^ MEF clone at intermediate and late passages resulted in a change from thymine to guanine which caused a substitution of valine to glycine at position 213 (Fig. 5b). A second guanine-to-cytosine mutation at nucleotide 403 could only be found in this late-passage *Wwox*^−/−^ MEF clone and led to an alanine-to-proline substitution at position 135 (Fig. 5c). Hotspot mutations at the consensus residues A138 and V216 in the DBD of human p53 have been observed in the cancer cells from patients (Fig. 5a) [67, 68]. No p53 mutations were observed in the control and other *Wwox*^−/−^ MEF clones. Although a large amount of the mutant p53 protein (A135P/V213G) was present in the nucleus of late-passage *Wwox*^−/−^ MEF clone no. 56, we detected a significantly decreased binding of nuclear p53 to the p53-response region (− 200 to + 98) at *p21*^*Cip1/Waf1*^ promoter by ChIP assay (Fig. 5d, e). We further analyzed whether the transcriptional activity of the endogenous p53 in late-passage *Wwox*^−/−^ MEF clone no. 56 is defective using a luciferase reporter plasmid carrying a p53-driven promoter. Similar to the finding of p21^Cip1/Waf1^ induction shown in Fig. 4c, we determined an increase in the transactivation activity of endogenous p53 in the control MEFs after serial passage culture (Fig. 5f). However, the p53-induced luciferase reporter activity was significantly reduced in late-passage *Wwox*^−/−^ MEF clone no. 56 (Fig. 5f). Ectopic overexpression of GFP-tagged wild-type p53 upregulated p21^Cip1/Waf1^, suppressed cell growth and induced cellular senescence in late-passage *Wwox*^−/−^ MEFs, as evidenced by western blotting, the use of a cell counting kit and SA-β-gal staining, respectively (Fig. 5g–i). As comparable levels of MDM2 were detected in MEFs of three genotypes, we determined a reduced binding of MDM2 to human mutant p53 protein (A138P/V216G), suggesting that p53 mutations at these residues may protect it from MDM2-mediated degradation (Supplementary Fig. 7). Collectively, our results suggest that functional inactivation of cellular p53 by either promoting its proteasomal and lysosomal degradation or loss-of-function mutations is prerequisite for suppressing p21^Cip1/Waf1^ expression, thereby leading to the senescence escape of late-passage *Wwox*^−/−^ MEFs.

### ROS suppression by antioxidant treatment leads to senescence induction in late-passage *Wwox*^−/−^ MEFs

**Fig. 6:**
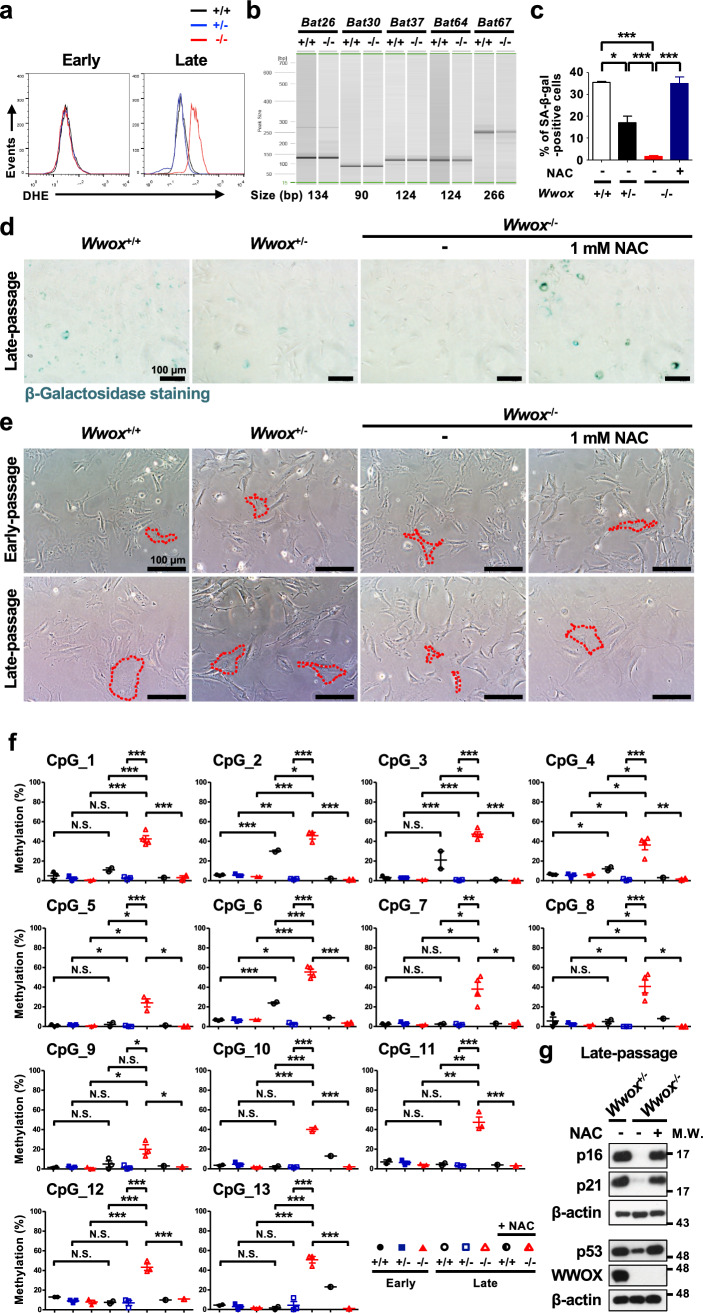
ROS inhibition results in senescence induction in late-passage *Wwox*^−/−^ MEFs. **a** ROS levels in *Wwox*^+/+^, *Wwox*^+/−^ and *Wwox*^−/−^ MEFs at early- and late-passages were determined by DHE staining and flow cytometric analysis. **b** PCR amplification of mononucleotide repeat markers *Bat26*, *Bat30*, *Bat37*, *Bat64* and *Bat67* was performed using the genomic DNA samples isolated from NAC-treated late-passage *Wwox*^+/+^ and *Wwox*^−/−^ MEFs, and the length of PCR products was analyzed by capillary electrophoresis for detecting microsatellite instability. **c**, **d** SA-β-gal staining was performed in late-passage *Wwox*^+/+^, *Wwox*^+/−^, *Wwox*^−/−^, and 1 mM NAC-treated *Wwox*^−/−^ MEFs. The representative images obtained from at least three independent experiments are shown in **d**. Scale bars = 100 µm. The percentages of SA-β-gal-positive senescent cells are shown in **c**. *P ≤ 0.05; ***P ≤ 0.005; Two-tailed *t* test. **e** Cellular morphology of *Wwox*^+/+^, *Wwox*^+/−^, *Wwox*^−/−^, and 1 mM NAC-treated *Wwox*^−/−^ MEFs at early- and late-passages was examined. Scale bars = 100 µm. **f** DNA methylation of *p16*^*Ink4a*^ promoter at specific CpG islands was analyzed. *N.S.* not significant; *P ≤ 0.05; **P ≤ 0.01; ***P ≤ 0.005; Two-tailed *t* test. **g** p16^Ink4a^, p21^Cip1/Waf1^, p53 and WWOX protein expression was examined in late-passage *Wwox*^+/−^, *Wwox*^−/−^, and 1 mM NAC-treated *Wwox*^−/−^ MEFs. β-actin was used as an internal control in western blotting

**Fig. 7:**
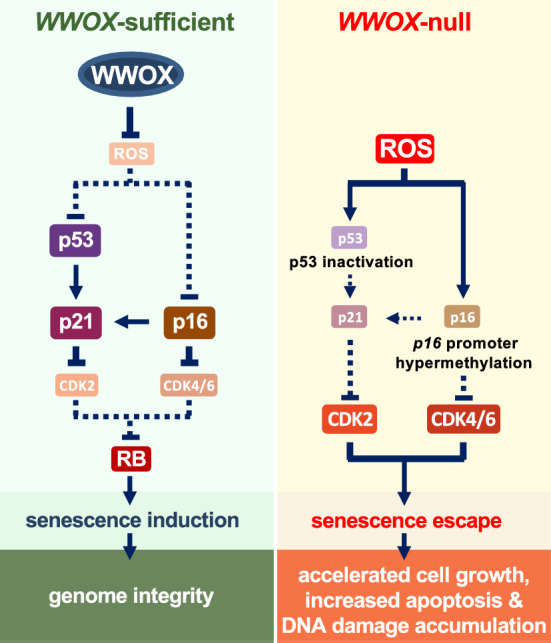
WWOX promotes senescence induction for maintaining genome integrity via inhibiting ROS overproduction in MEFs. In WWOX-null MEFs, excessive ROS accumulation during in vitro passage culture causes p53 mutations and protein destabilization and *p16*^*Ink4a*^ promoter hypermethylation. Significant downregulation of CDK inhibitors, including p21^Cip1/Waf1^ and p16^Ink4a^, leads to senescence escape, increased cell proliferation and apoptosis, and genome instability in late-passage *Wwox*^−/−^ MEFs

We have previously shown that WWOX translocates to the mitochondria via its SDR domain in response to stress [69, 70]. Drosophila WWOX has been suggested to regulate aerobic metabolism, mitochondrial respiratory complexes and endogenous ROS generation [71, 72]. In spite of the fact that ROS generation is a major determinant of cellular senescence [8, 73, 74], intracellular accumulation of excessive oxidative damages triggered by dramatically increased ROS may lead to epigenetic and gene expression changes, genome instability and uncontrolled cell growth. By microarray analysis, transcriptional induction of a series of genes that participate in redox homeostasis was observed in the newly isolated MEFs from *Wwox*^−/−^ mouse embryos (Supplementary Fig. 8), suggesting that *Wwox* deficiency may result in a substantial redox perturbation in cells. By DHE staining and flow cytometric analysis, we detected a substantially increased level of ROS in late-passage *Wwox*^−/−^ MEFs (Fig. 6a). To test whether the increased ROS generation due to *Wwox* deficit confers the defective senescence signaling, MEFs were continually maintained in the medium supplemented with antioxidant NAC after isolation from mouse embryos. Interestingly, treatment of cells with NAC during the passage culture prevented microsatellite instability and resulted in senescence induction in the late-passage *Wwox*^−/−^ MEFs, as evidenced by their increased SA-β-gal activity and morphological changes (Fig. 6b–e). By quantitative analysis of DNA methylation spanning specific CpG islands, *p16*^*Ink4a*^ promoter hypermethylation in late-passage *Wwox*^−/−^ MEFs was blocked by NAC treatment (Fig. 6f). The downregulation of p16^Ink4a^, p21^Cip1/Waf1^ and p53 protein expression in late-passage *Wwox*^−/−^ MEFs was reversed by the presence of NAC (Fig. 6g). In summary, our findings clearly indicate a crucial role of WWOX in the regulation of p16^Ink4a^ and p21^Cip1/Waf1^ signaling and induction of replicative senescence for maintaining genome integrity through inhibiting ROS overproduction in MEFs (Fig. 7).

## Discussion

We have established ex vivo MEF passage cultures and demonstrated that WWOX maintains cellular genome integrity under DNA replication stress through induction of cellular senescence. Without WWOX, aberrant duplication of genetic information may cause genome instability and accumulation of deleterious mutations, thereby leading to uncontrolled cell division or death. Most intriguingly, we determined that WWOX blocks ROS-mediated downregulation of p16^Ink4a^ and p21^Cip1/Waf1^ for triggering cellular senescence. Human *WWOX* gene resides at the chromosomal fragile site *FRA16D* that exhibits an increased frequency of breakage upon replication stress and is preferentially unstable during cancer progression [18]. Mutations, deletions, loss of heterozygosity, and translocations of *WWOX* gene have been noted in many types of human cancers [40]. Unfavorable clinical outcomes in patients are associated with reduced or absent expression of WWOX in cancer specimens [75]. The loss of WWOX expression in hyperplastic or cancer cells upon DDR induction may lead to senescence escape and consequently promote aberrant cell proliferation and genome instability. The resulting genetic alterations accumulate and may include inactivation of tumor suppressors, such as p53, and oncogene amplification and activation that are intimately linked to the proliferative capacity of cells and drive aggressive tumors.

Cellular senescence occurs throughout the life and plays important roles in a variety of physiology and pathological processes, including embryonic development, wound healing, fibrosis, inflammation, ageing, prevention of neoplastic transformation, Alzheimer's disease, atherosclerosis, and type 2 diabetes [3, 6, 8, 14, 76]. Early lethal microcephaly syndrome with epilepsy, neurodegeneration and profound growth retardation have been reported in the pediatric patients with autosomal recessive homozygous mutation of *WWOX* [33–38]. Whole-body *Wwox*^−/−^ mice succumb to severe neurodevelopmental deficits, impaired hematopoiesis and metabolic disorders, and survive for less than a month [31, 32, 77]. Recent studies have clearly illustrated the connections of defective DDR machinery to congenital neuropathies, neurodegenerative diseases and metabolic dysfunction [78, 79]. Whether genome instability resulting from dysregulation of the DDR pathways and senescence escape in the cells lacking WWOX interferes with cell physiology and homeostasis of systemic metabolism by causing aberrant cell proliferation or apoptosis in patients and animals remains to be verified. Excessive oxidative damages of cellular macromolecules and supramolecular complexes or activation of specific signaling pathways resulting from overproduction of intracellular ROS in WWOX-null cells may also lead to the deleterious effects and early lethality in patients with loss-of-function mutation in *WWOX* gene.

Mitochondria play a vital role to orchestrate cellular energy production through oxidative phosphorylation and are a major source of intracellular ROS [74]. A previous study has shown that loss of WWOX expression promotes glycolysis and diminishes mitochondrial respiration, suggesting that WWOX may regulate metabolic reprogramming and redox homeostasis in cells [80]. WWOX deficiency exacerbates the phenotypic alterations in the developing *Drosophila* eye due to cellular dysfunction caused by decreased expression of mitochondrial respiratory chain components [71]. The cellular dysfunction engendered by knockdown of a mitochondrial respiratory complex gene *ND42* in larval eye discs is associated with increased ROS and could be rescued by overexpression of antioxidant enzymes [71]. Stringent regulation of intracellular ROS levels is crucial for cell fate determination. High ROS levels cause oxidative damage and cell death, whereas low concentrations of ROS may be required for supporting normal cell homeostasis and growth [81, 82]. WWOX possesses a short-chain alcohol dehydrogenase/reductase domain at its C-terminus and has been suggested to regulate intracellular ROS levels in *Drosophila* larvae [72]. Although no apparent differences in the intracellular ROS levels were detected between the early-passage control and *Wwox*^−/−^ MEFs, we determined significant increases in the induction of redox-regulatory genes, ROS scavengers in particular, in the freshly isolated *Wwox*^−/−^ MEFs. Whether perturbation of redox homeostasis due to *Wwox* loss in early-passage MEFs triggers an initial antioxidative response necessary to buffer the rising ROS and mitigate cellular damage until the scavenging machinery becomes ineffective as a result of excessive ROS accumulation beyond a threshold after passage culture is unclear. Also, the molecular mechanism by which WWOX controls intracellular ROS generation needs further investigation.

ROS overproduction in cells may exhaust the cellular antioxidant capacity and has been suggested to cause deleterious DNA lesions and participate in epigenetic processes, including DNA methylation and histone methylation and acetylation, for altering gene expression [83, 84]. Hypermethylation of *p16*^*Ink4a*^ gene promoter has been shown to be induced by oxidative stress and promote cancer progression in human esophageal adenocarcinoma [85]. We determined that treatment of either 5-Aza or NAC reversed the reduction in protein expression of both p16^Ink4a^ and p21^Cip1/Waf1^ in late-passage *Wwox*^−/−^ MEFs, suggesting the involvement of ROS-mediated promoter methylation in the CDK inhibitor downregulation in *Wwox*-deficient cells during the passage culture. Using a bisulfite conversion approach, we demonstrated that NAC treatment prevented *p16*^*Ink4a*^ promoter hypermethylation in late-passage *Wwox*^−/−^ MEFs. Nevertheless, the methylation status of *p21*^*Cip1/Waf1*^ promoter at specific CpG sites showed no significant differences between the control and *Wwox*^−/−^ MEFs (Supplementary Fig. 9), indicating that the p21^Cip1/Waf1^ downregulation in *Wwox*^−/−^ MEFs is independent of its promoter methylation. Ectopic p16^Ink4a^ increased p21^Cip1/Waf1^ expression in late-passage *Wwox*^−/−^ MEFs (lane 3 in Fig. 3c), suggesting that p16^Ink4a^ may act as an upstream effector to trigger p21^Cip1/Waf1^ induction. It is possible that 5-Aza treatment induces p16^Ink4a^ upregulation, which in turn stimulates p21^Cip1/Waf1^ expression in the late-passage *Wwox*^−/−^ MEFs. Therefore, both *p16*^*Ink4a*^ promoter hypermethylation and p53 deficit or dysfunction contribute to p21^Cip1/Waf1^ downregulation in *Wwox*-deficient MEFs (Fig. 7). How p16^Ink4a^ cooperates with p53 to regulate p21^Cip1/Waf1^ expression in MEFs is unclear.

When sensing the presence of DNA damage or stalled replication fork, CDK inhibitors are induced to arrest cell cycle progression to allow time for repair before the DNA lesion is passed on to daughter cells. Permanent proliferative arrest of cells caused by persistent DDR signaling due to DNA damage accumulation or progressive telomere erosion may lead to cellular senescence. After serial passage culture, increased levels of γH2AX and CDK inhibitors p16^Ink4a^, p21^Cip1/Waf1^ and p27^Kip1^ as well as senescence induction were observed in Wwox-sufficient control MEFs, suggesting that continuous in vitro cell replication may cause genome instability and induce DDR signaling in MEFs. ITCH-mediated ubiquitination of WWOX after DNA damage has been shown to be associated with the activation of checkpoint kinases ATM and ATR for induction of DDR signaling [26, 27]. WWOX also supports non-homologous end-joining repair of DNA double-strand breaks via interacting with Brca1 [29]. These findings are in agreement with the role of WWOX as a genome caretaker [28]. Without WWOX, successive accumulation of genetic and epigenetic alterations resulting from senescence escape and accelerated cell cycle progression may exacerbate genome instability further in cells with defective DNA repair pathways. The accumulative aberrations may also have a profound impact on cellular signaling and functions and render the WWOX-deficient cells vulnerable to apoptosis.

Many WW domain-containing proteins have a functional role in the ubiquitin–proteasome system [86]. Tyr33-phosphorylated WWOX physically interacts with and stabilizes p53 during tumor necrosis factor, ultraviolet light, and staurosporine-mediated stress responses [87]. We detected downregulation of p53 protein expression in the late-passage *Wwox*^−/−^ MEF clones except the clone no. 56 harboring mutant p53 (A135P/V213G). It is possible that WWOX blocks protein degradation of wild-type p53 via preventing its binding to the E3 ubiquitin ligase MDM2 [87]. The A135P/V213G mutations in p53 may protect it from degradation in WWOX-deficient cells as a result of the reduced binding of this mutant p53 protein to MDM2. Whether WWOX promotes p27^Kip1^ protein stability through inhibiting its phosphorylation at Thr187 and/or ubiquitination by SCF^Skp2^ is unclear.

Cellular senescence has emerged as an important contributor to tumor suppression, aging and many age-related diseases [2]. Targeting WWOX can be exploited in the treatment of senescence-related diseases. Delineation of WWOX-regulated signaling pathways upon DDR activation will gain a greater understanding of the pathogenesis of cancers, age-related diseases and early postnatal lethality due to WWOX loss in humans. The establishment of murine models with tissue-specific knockout for *Wwox* gene may help us unravel the in vivo function of WWOX during disease development.

### Supplementary Information

Below is the link to the electronic supplementary material.

Supplementary file1 (PDF 3511 KB)

## Data Availability

The authors declare that all data supporting the findings of this study are available within the article and its supplementary information files.
