# Supplementary material for: Loss of fragile WWOX gene leads to senescence escape and genome instability
Source: Cell Mol Life Sci. 2023 Oct 28;80(11):338. doi: 10.1007/s00018-023-04950-1 (PMC10613160; doi:10.1007/s00018-023-04950-1)
Supplement: Supplementary file 1 — Supplementary file1 (PDF 3511 KB) [file 18_2023_4950_MOESM1_ESM.pdf]

Loss of fragile *WWOX* gene leads to senescence escape and genome instability

Hui-Ching Cheng[1], Po-Hsien Huang[2,†], Feng-Jie Lai[3,4,†], Ming-Shiou Jan[5,†], Yi-Lin Chen[6,7], Szu-Ying Chen[2], Wan-Li Chen[7], Chao-Kai Hsu[8], Wenya Huang[6], Li-Jin Hsu[1,6,9,10]

[1]Institute of Basic Medical Sciences, College of Medicine, National Cheng Kung University, Tainan 70101, Taiwan

[2]Department of Biochemistry and Molecular Biology, College of Medicine, National Cheng Kung University, Tainan 70101, Taiwan

[3]Department of Dermatology, Chi Mei Medical Center, Tainan 71004, Taiwan

[4]Center for General Education, Southern Taiwan University of Science and Technology, Tainan 71005, Taiwan

[5]Institute of Biochemistry, Microbiology and Immunology, Chung Shan Medical University, Taichung 40201, Taiwan

[6]Department of Medical Laboratory Science and Biotechnology, College of Medicine, National Cheng Kung University, Tainan 70101, Taiwan

[7]Molecular Diagnosis Laboratory, Department of Pathology, National Cheng Kung University Hospital, Tainan 704302, Taiwan

[8]Department of Dermatology, College of Medicine, National Cheng Kung University, Tainan 70101, Taiwan

[9]Center of Infectious Disease and Signaling Research, College of Medicine, National Cheng Kung University, Tainan 70101, Taiwan

[10]Research Center for Medical Laboratory Biotechnology, College of Medicine, National Cheng Kung University, Tainan 70101, Taiwan

[†]Po-Hsien Huang, Feng-Jie Lai and Ming-Shiou Jan contributed equally to this work.

Correspondence

Li-Jin Hsu, Department of Medical Laboratory Science and Biotechnology, College of Medicine, National Cheng Kung University, Tainan 70101, Taiwan.

Email: ljhsu@mail.ncku.edu.tw; hsu.lijin@gmail.com

Feng-Jie Lai, Department of Dermatology, Chi Mei Medical Center, Tainan 71004, Taiwan.

Email: lai.fengjie@gmail.com

Supplementary Table 1. Primer and shRNA sequences

Target gene	Primer sequences	Size (b.p.)
**Reverse transcription-PCR**		
Mouse *β-actin*	Forward: 5'-TGGAATCCTGTGGCATCCATGAAAC<br>Reverse: 5'-TAAAACGCAGCTCAGTAACAGTCCG	349
Mouse *Arf*	Forward: 5'-GCCGCACCGGAATCCT<br>Reverse: 5'-TTGAGCAGAAGAGCTGCTACGT	67
Mouse *p15*	Forward: 5'-AGATCCCAACGCCCTGAAC<br>Reverse: 5'-CCCATCATCATGACCTGGATT	57
Mouse *p16*	Forward: 5'-CGTACCCCGATTCAGGTGAT<br>Reverse: 5'-TTGAGCAGAAGAGCTGCTACGT	59
Mouse *p18*	Forward: 5'-ACGTCAACGCTCAAAATGGA<br>Reverse: 5'-TAGCACCTCTGAGGAGAAGCCT	96
Mouse *p19*	Forward: 5'-TGAACCGCTTTGGCAAGAC<br>Reverse: 5'-ACTAGTACCGGAGGCATCTTGG	116
Mouse *p21*	Forward: 5'-TTGCACTCTGGTGTCTGAGC<br>Reverse: 5'-TCTGCGCTTGGAGTGATAGA	112
Mouse *p27*	Forward: 5'-TTGGGTCTCAGGCAAACTCT<br>Reverse: 5'-TCTGTTCTGTTGGCCCTTTT	157
Mouse *p53*	Forward: 5'-TGGAGGAGTCACAGTCGGATA<br>Reverse: 5'-GTCCATGCAGTGAGGTGATG	116
Mouse *p57*	Forward: 5'-GCGCAAACGTCTGAGATGAGT<br>Reverse: 5'-AGAGTTCTTCCATCGTCCGCT	88
Mouse *Wwox*	Forward: 5'-ACTACGCCAATCACACTGAGG<br>Reverse: 5'-GTCCACGGTAAATGCCAATC	188
**ChIP assay**		
Mouse *p21* promoter [-200, +98]	Forward: 5'-CCTTTCTATCAGCCCCAGAGGATACC<br>Reverse: 5'-GGGACGTCCTTAATTATCTGGGGTC	299
**Mouse *p53* cDNA sequencing**		
p53 [-101, +663]	Forward: 5'-CCATCCTGGCTGTAGGTAGC<br>Reverse: 5'-CTCGGGTGGCTCATAAGGTA	764
p53 [+577, +1228]	Forward: 5'-CGGGTGGAAGGAAATTTGTA<br>Reverse: 5'-TATGGCGGGAAGTAGACTGG	652
**Gender typing**		
Mouse *Ube1*	Forward: 5'-TGGTCTGGACCCAAACGCTGTCCACA<br>Reverse: 5'-GGCAGCAGCCATCACATAATCCAGATG	211/189
Mouse *Sry*	Forward: 5'-CTGTGTAGGATCTTCAATCTCT<br>Reverse: 5'-GTGGTGAGAGGCACAAGTTGGC	148
Mouse *Zfy*	Forward: 5'-GACTAGACATGTCTTAACATCTGTCC<br>Reverse: 5'-CCTATTGCATGGACTGCAGCTTATG	184

**Microsatellite instability assay**		
Mouse *Bat26*	Forward: 5'-TCACCATCCATTGCACAGTT<br>Reverse: 5'-CTGCGAGAAGGTACTCACCC	134
Mouse *Bat30*	Forward: 5'-ATTTGGCTTTCAAGCATCCATA<br>Reverse: 5'-GGGAAGACTGCTTAGGGAAGA	90
Mouse *Bat37*	Forward: 5'-TCTGCCCAAACGTGCTTAAT<br>Reverse: 5'-CCTGCCTGGGCTAAAATAGA	124
Mouse *Bat64*	Forward: 5'-GCCCACACTCCTGAAAACAGTCAT<br>Reverse: 5'-CCCTGGTGTGGCAACATTAAGC	124
Mouse *Bat67*	Forward: 5'-CCGACTGCTCTTCCGAAGGTC<br>Reverse: 5'-TTGCCCATTTATCATCTAGTTCAT	266
**EpiTYPER analysis**		
*CDKN1A*oPra1 [29310071, 29309800]	Forward: 5'-AGGAAGAGAGTTTTATTTTGTTGGTAAAGTGGGA<br>Reverse: 5'-CAGTAATACGACTCACTATAGGGAGAAGGCTCCCATCCCTAACTATTACCTCTC	272
*CDKN2A*oPra1 [89200195, 89200456]	Forward: 5'-AGGAAGAGAGGATTGTAGATGGGATATTTTTTGTTTATTTG<br>Reverse: 5'-CAGTAATACGACTCACTATAGGGAGAAGGCTATCCTCAAAAAAAAAAAAAAAAAACCCAC	262
*CDKN2A*oPra2 [89200541, 89200813]	Forward: 5'-AGGAAGAGAGGTATGAATTGAATATTTTTGAAAATATTGTTG<br>Reverse: 5'-CAGTAATACGACTCACTATAGGGAGAAGGCTAAAAAAACTAATTCACTTTCTCAAAAAACAC	273
**Construction**		
pcDNA3/ HA-tagged mouse *p16*	Forward:<br>5'-AAATTTGGATCCATGGAGTCCGCTGCAGAC<br>Reverse:<br>5'-GGGCTCGAATTCTTAGCTCTGCTCTTGGGA	531
**Site-directed mutagenesis**		
pEGFPN1/ Human p53(R72P)	Forward:<br>5’-CCAGAGGCTGCTCCCCCCGTGGCCCCTGCA<br>Reverse:<br>5’-TGCAGGGGCCACGGGGGGAGCAGCCTCTGG	
pEGFPN1/ Human p53(A138P)	Forward:<br>5’-TTTTGCCAACTGCCCAAGACCTGCCCTGTG<br>Reverse:<br>5’-CACAGGGCAGGTCTTGGGCAGTTGGCAAAA	
pEGFPN1/	Forward:<br>5’-ACTTTTCGACATAGTGGGGTGGTGCCCTATGAG	

Human p53(V216G)	Reverse: 5'-CTCATAGGGCACCACCCCACTATGTCGAAAAGT	
**Target gene**	**Oligo sequences**	**Clone ID**
**shRNA**		
Human *WWOX*	5'-CCGGGCCAAGAATGTGCCTCTTCATCTCGAGATGAAGAGGCACATTCTTGGCTTTTTG	TRCN0000033840
Luciferase	5'-CCGGGCGGTTGCCAAGAGGTTCCATCTCGAGATGGAACCTCTTGGCAACCGCTTTTTG	TRCN0000072249

Supplementary Figure 1

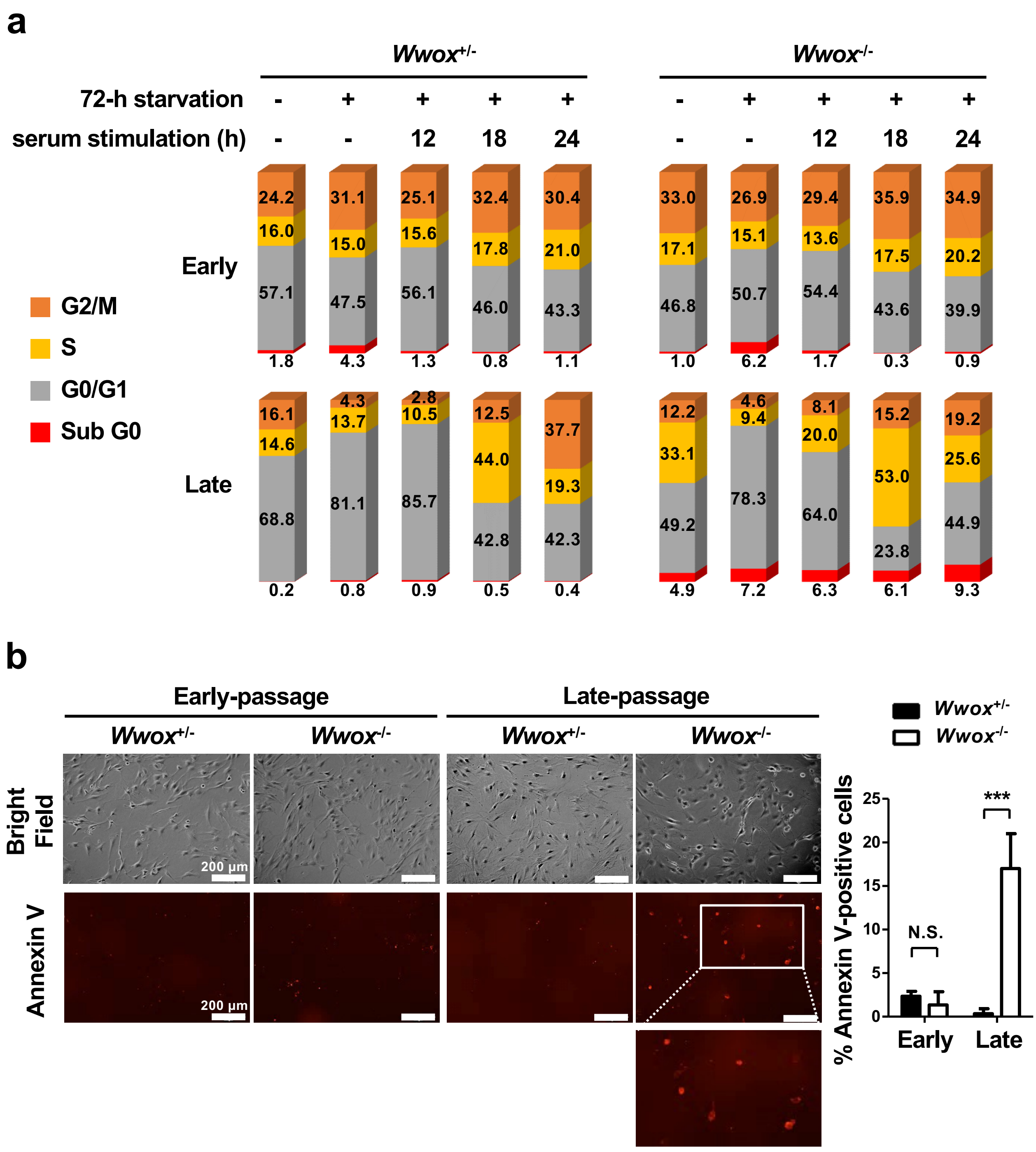

c

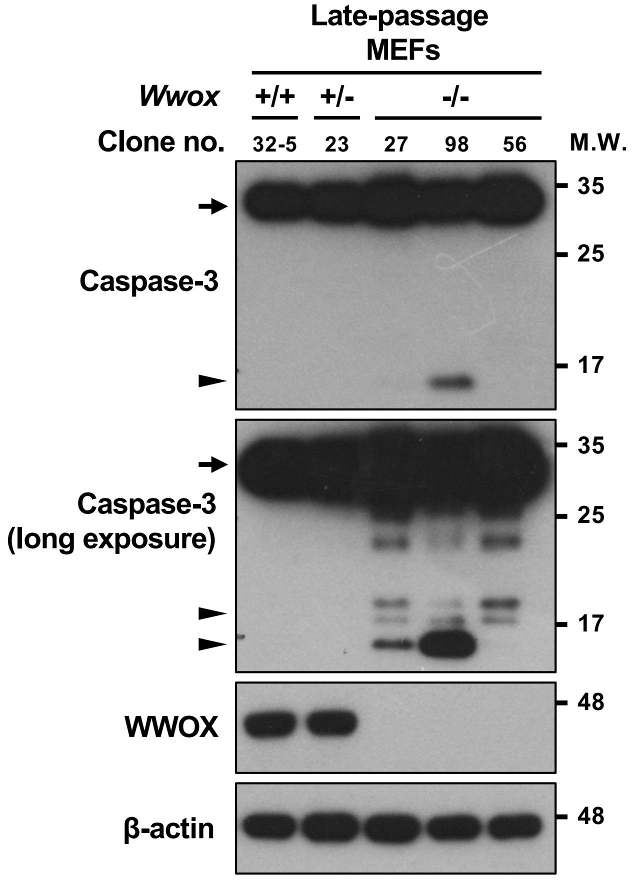

Fig. S1 Aberrant cell proliferation and apoptosis in late-passage *Wwox*$^{-/-}$ MEFs. **a** *Wwox*$^{+/-}$ and *Wwox*$^{-/-}$ MEFs at early- and late-passages were cultured in a low serum condition (0.1% fetal bovine serum in DMEM) for 72 h for synchronization. After serum starvation, MEFs were treated with 10% fetal bovine serum and incubated for an additional 12, 18 or 24 h. After fixing the cells with 70% ethanol/PBS, cell cycle analysis by propidium iodide staining and flow cytometry was performed. The percentages of cells in sub G0, G0/G1, S and G2/M are shown. **b** Apoptotic cells were detected using phycoerythrin-conjugated annexin V in *Wwox*$^{+/-}$ and *Wwox*$^{-/-}$ MEFs at early- and late-passages. Representative images obtained from at least three independent experiments are shown. The percentages of annexin V-positive cells (red) are shown in the right panel. Data are presented as mean ± standard deviation (SD). Scale bars = 200 μm. N.S., not significant; ***, $P \leq 0.005$; One-way ANOVA. **c** The increases of cleaved caspase-3 (arrowheads) were detected in *Wwox*$^{-/-}$ MEFs at late-passages by western blotting. Arrows indicate procaspase-3. β-actin was used as an internal control.

Supplementary Figure 2

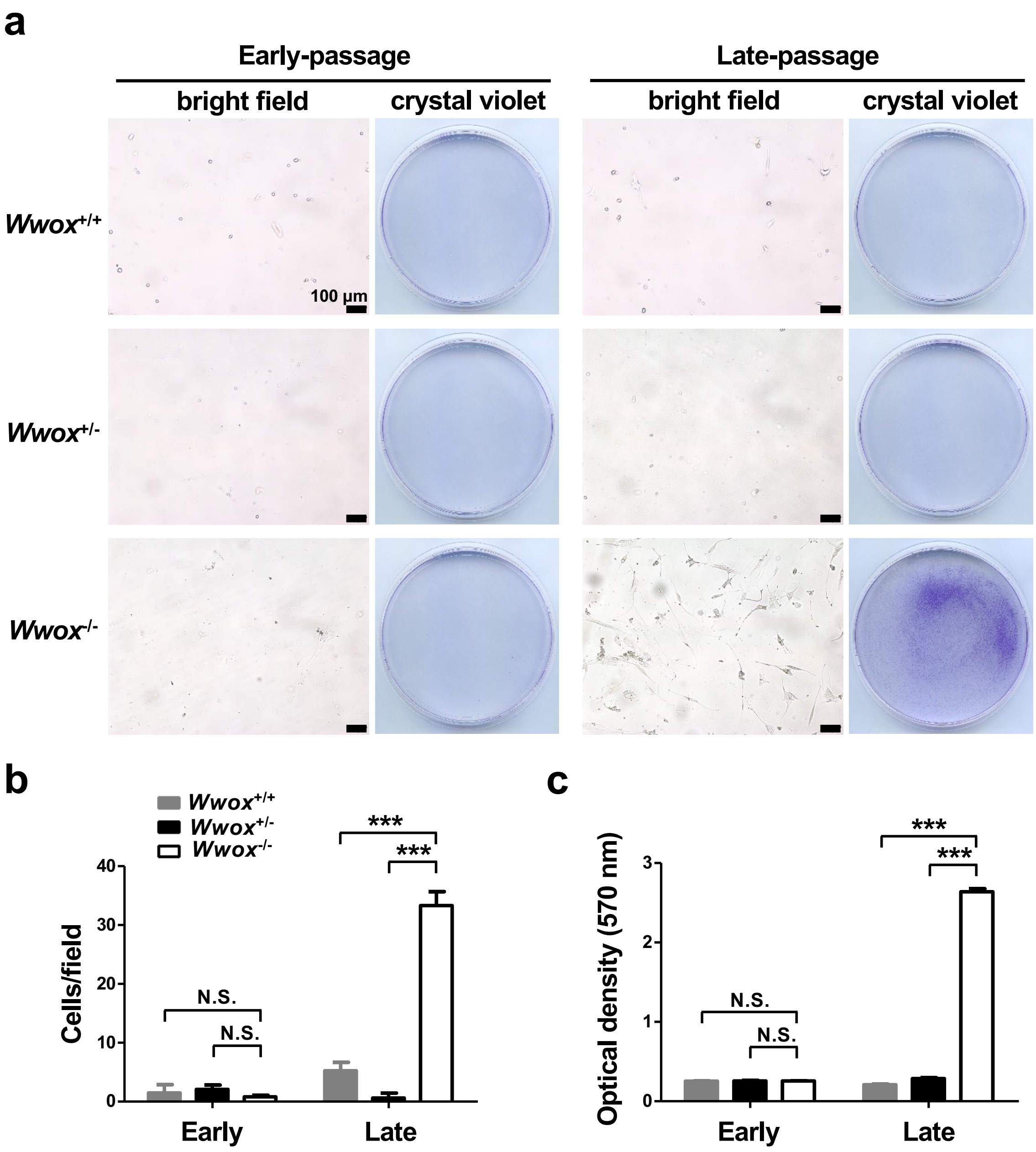

d

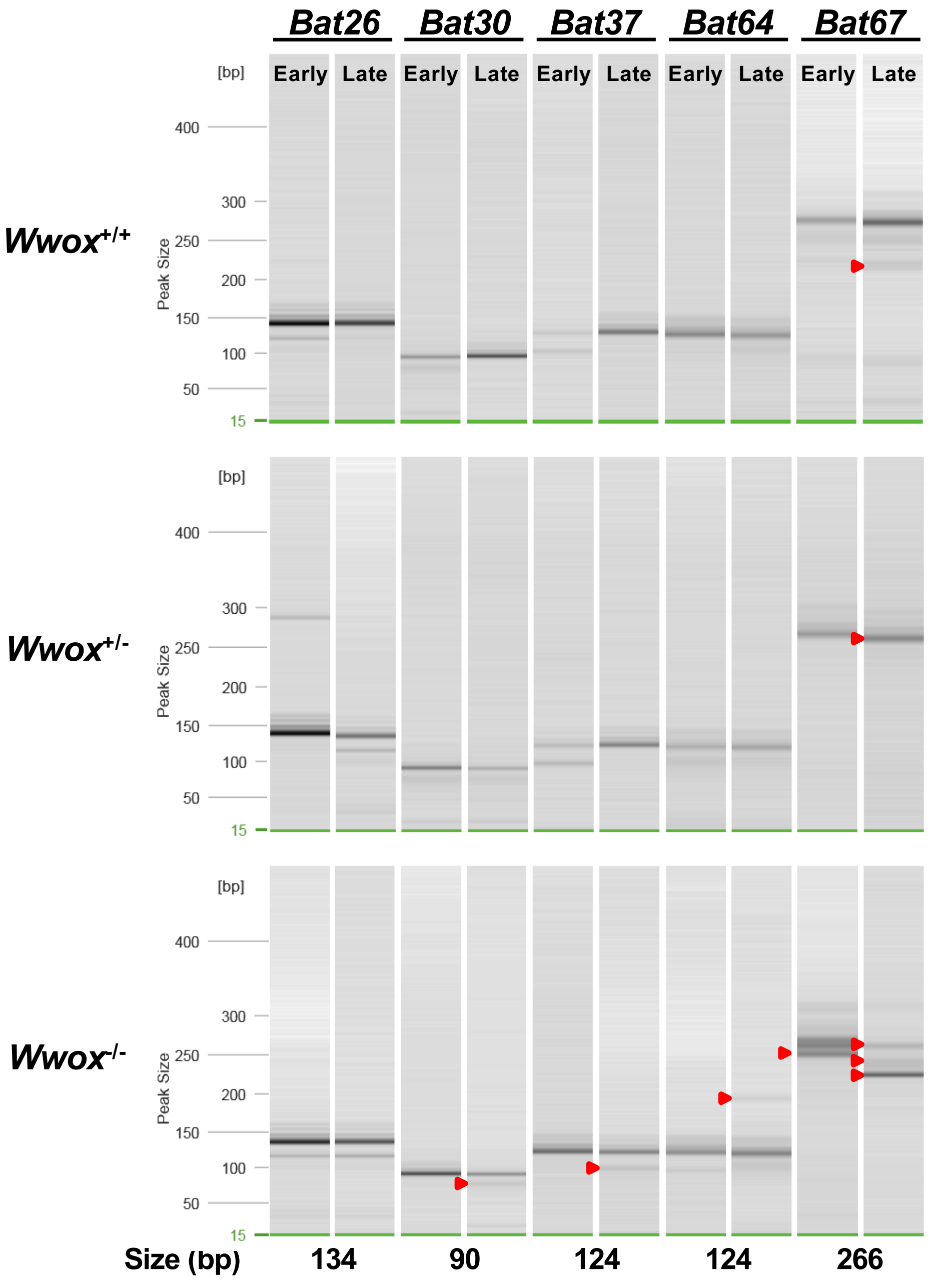
Bat26
Bat30
Bat37
Bat64
Bat67
Early
Late
[bp]
400
300
250
200
150
100
50
15
Peak Size
Wwox+/+
Wwox+/-
Wwox-/-
Size (bp)
134
90
124
124
266

Fig. S2 Increased DNA instability in late-passage *Wwox*$^{-/-}$ MEFs. **a**-**c** 6-Thioguanine resistance acquired by X-linked *Hprt* gene mutations was analyzed in 6-thioguanine-treated *Wwox*$^{+/+}$, *Wwox*$^{+/-}$ and *Wwox*$^{-/-}$ MEFs at early- and late-passages as described in Materials and Methods. After removal of detached dead cells, the 6-thioguanine-resistant MEFs on culture dishes were stained with crystal violet and examined under a light microscope (**a**), and the cell numbers per power field (10x) were counted (**b**). The cell-bound crystal violet was dissolved with acetic acid solution and quantified by measuring the absorbance of the eluates using a microplate reader set at 570 nm (**c**). Data are presented as mean ± SD. Scale bars = 100 μm. N.S., not significant; ***, P ≤ 0.005; Two-tailed *t* test. **d** Increased microsatellite DNA instability in late-passage *Wwox*$^{-/-}$ MEFs. The genomic DNA samples isolated from *Wwox*$^{+/+}$, *Wwox*$^{+/-}$ and *Wwox*$^{-/-}$ MEFs at early- and late-passages were used for PCR amplification of mononucleotide repeat markers *Bat26*, *Bat30*, *Bat37*, *Bat64* and *Bat67*. The size of PCR products was analyzed by capillary electrophoresis. The red arrowheads indicate the changes in the length of microsatellite repetitive DNA sequences in MEFs.

Supplementary Figure 3

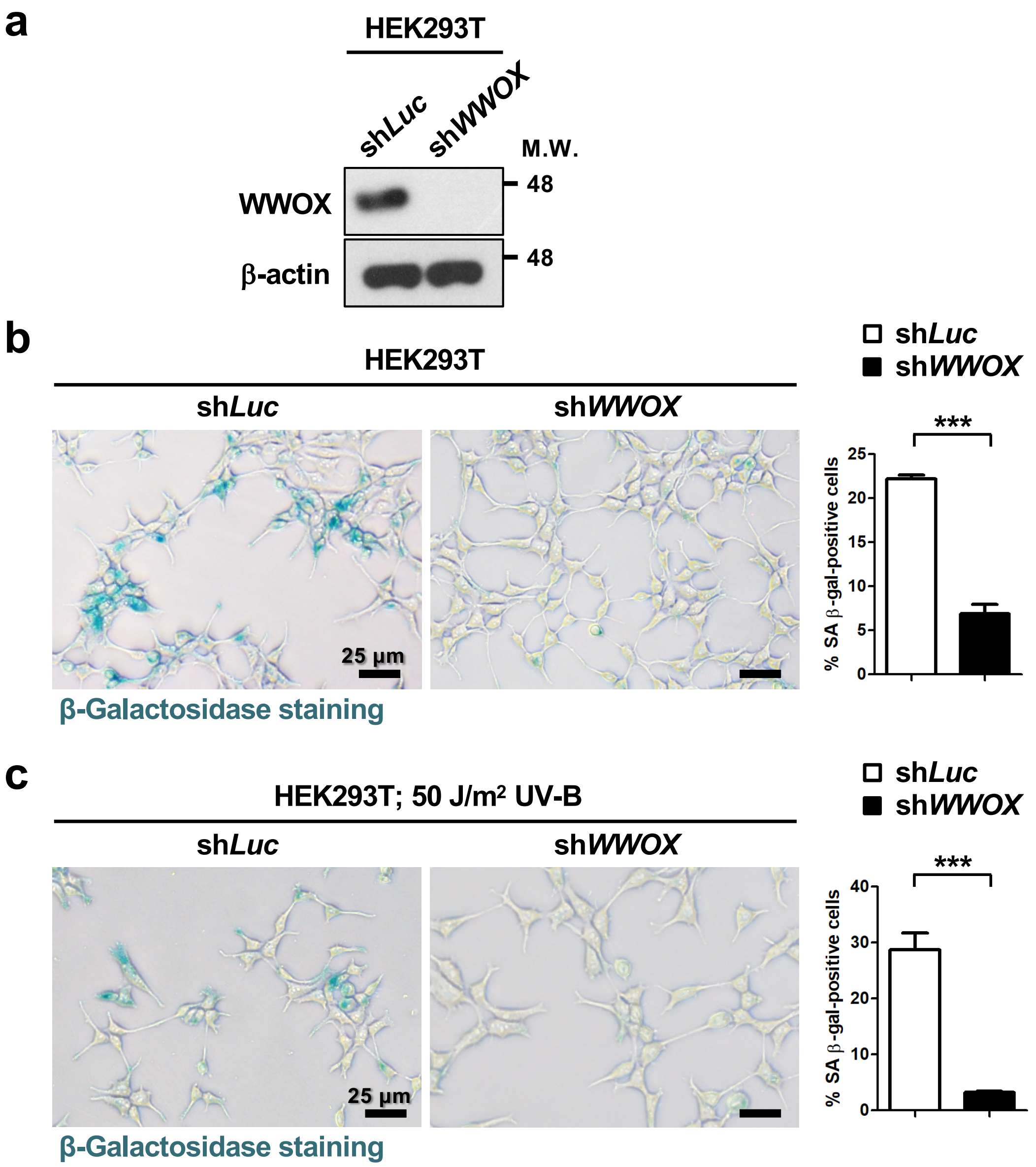

Fig. S3 *WWOX*-knockdown decreases senescence induction in HEK293T cells. **a** Lentivirus-mediated knockdown of WWOX was performed in HEK293T cells. WWOX protein expression in control (sh*Luc*) and WWOX-knockdown cells (sh*WWOX*) was detected by western blotting. β-actin was used as an internal control. **b** Cellular senescence was examined in control and WWOX-knockdown HEK293T cells by SA-β-gal staining after multiple passages. Representative images obtained from at least three independent experiments are shown. The percentages of SA-β-gal-positive cells are shown in the right panel. Data are presented as mean ± SD. Scale bars = 25 μm. **c** Control and WWOX-knockdown HEK293T cells were exposed to 50 J/m^2 ultraviolet (UV)-B, incubated at 37℃ for 5 days, and examined for senescence induction by SA-β-gal staining. Scale bars = 25 μm. ***, $P \leq 0.005$; One-way ANOVA.

Supplementary Figure 4

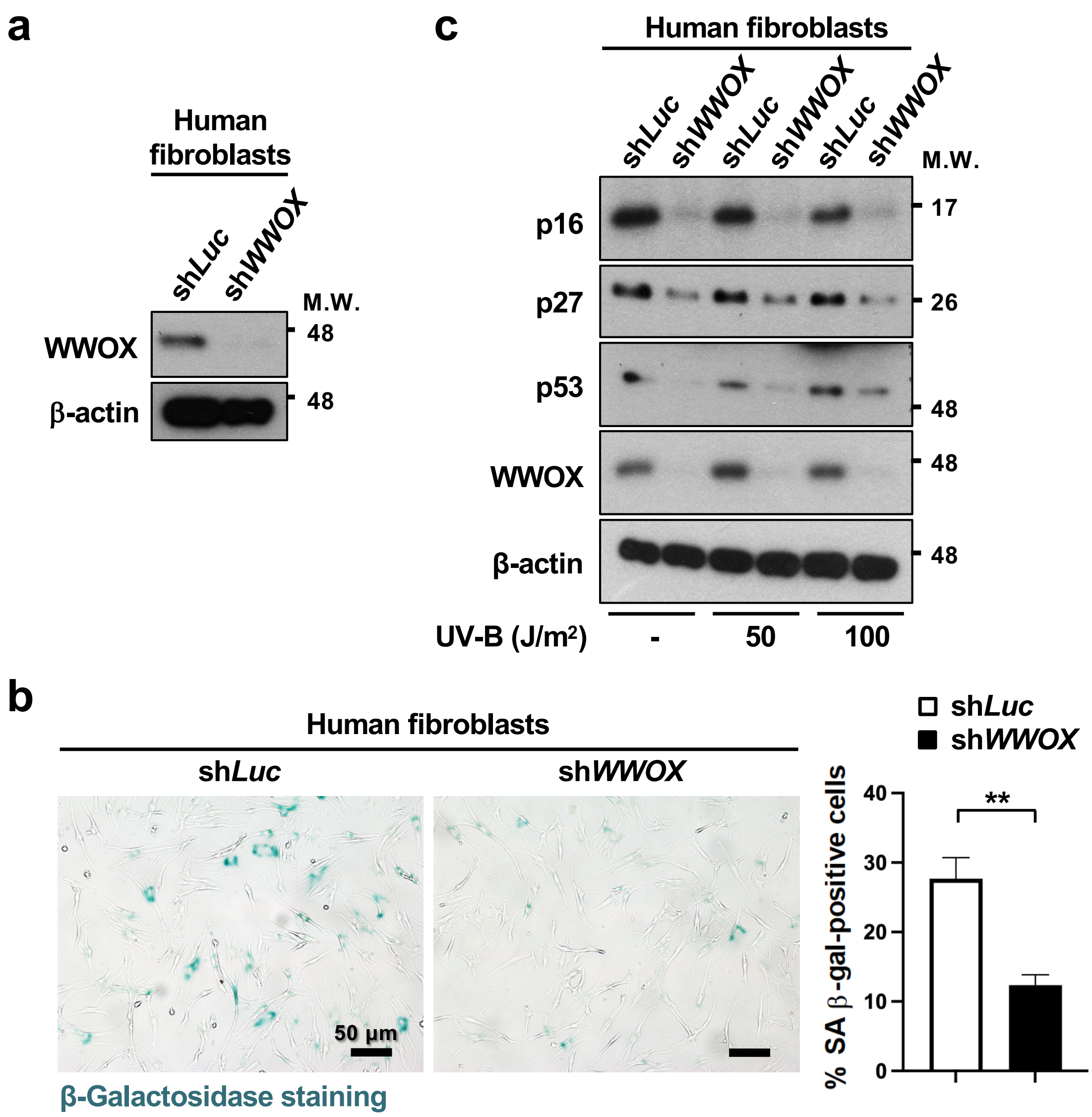

Fig. S4 *WWOX*-knockdown decreases senescence induction in human skin fibroblasts. **a** Lentivirus-mediated knockdown of WWOX was performed in primary fibroblasts isolated from human skin. WWOX protein expression in control (sh*Luc*) and WWOX-knockdown cells (sh*WWOX*) was detected by western blotting. β-actin was used as an internal control. **b** Cellular senescence was examined in control and WWOX-knockdown human fibroblasts by SA-β-gal staining after multiple passages. Representative images obtained from at least three independent experiments are shown. The percentages of SA-β-gal-positive cells are shown in the right panel. Data are presented as mean ± SD. Scale bars = 50 μm. **, P ≤ 0.01; One-way ANOVA. **c** The human sh*Luc* and sh*WWOX* fibroblasts were treated with the indicated doses of UV-B, and then incubated at 37℃ for 5 days. The expression levels of $p16^{Ink4a}$, $p27^{Kip1}$, p53, WWOX and β-actin proteins in cells were examined by western blotting.

Supplementary Figure 5

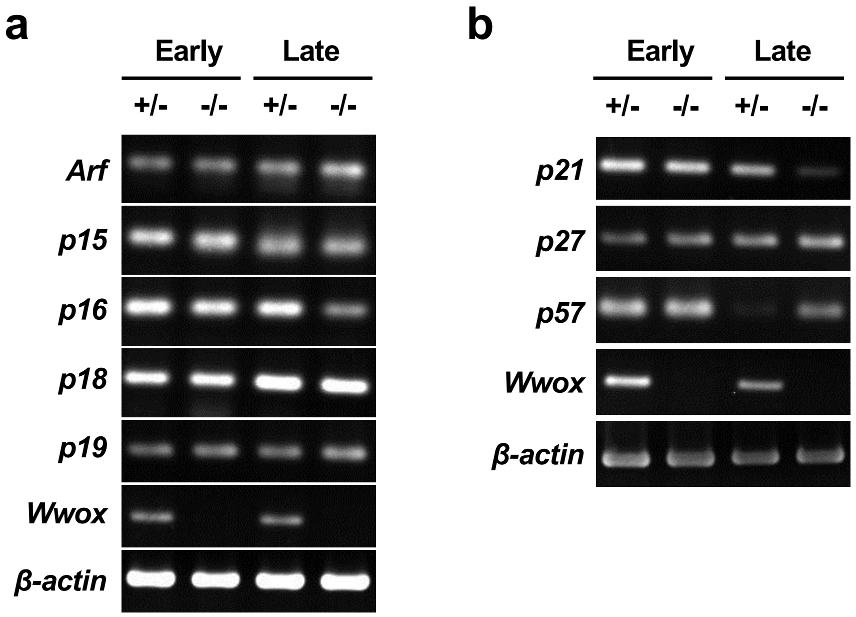

Fig. S5 Decreased mRNA levels of CDK family genes *p16*^{Ink4a} and *p21*$^{Cip1/Waf1}$ in late-passage *Wwox*$^{-/-}$ MEFs. Using the RNA isolated from *Wwox*$^{+/-}$ and *Wwox*$^{-/-}$ MEFs at early- and late-passages, reverse transcription was performed to generate complementary DNA samples for PCR. The amplification products using the specific primer pairs listed in Supplementary Table 1 were separated by agarose gel electrophoresis and visualized to assess the expression of INK (**a**) and KIP/CIP (**b**) CDK family genes. β-actin was used as an internal control.

Supplementary Figure 6

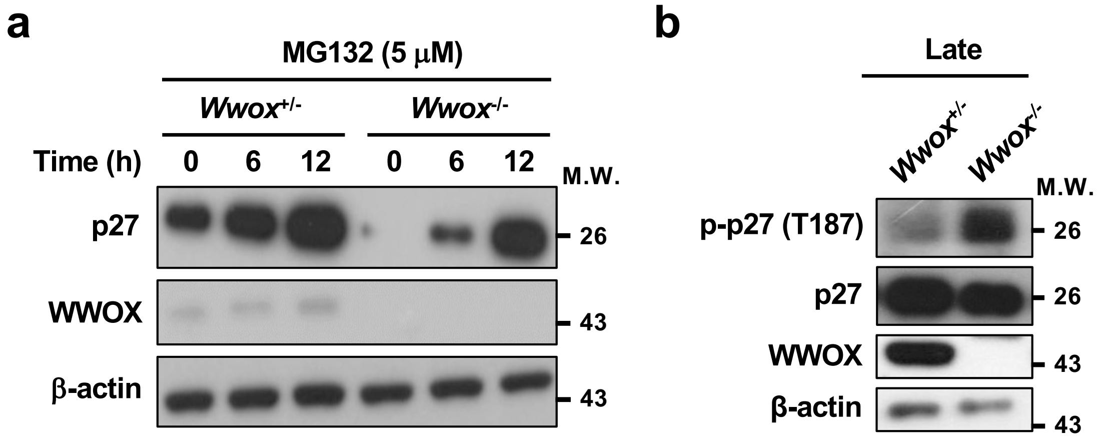

Fig. S6 Increased phosphorylation and proteasomal degradation of p27^{Kip1} protein in late-passage *Wwox*$^{-/-}$ MEFs. **a** Late-passage *Wwox*$^{+/-}$ and *Wwox*$^{-/-}$ MEFs were treated with a proteasome inhibitor MG132 (5 μM) for the indicated time periods. The protein expression of p27^{Kip1} and WWOX was determined by western blotting. β-actin was used as an internal control. **b** Protein expression levels of p27^{Kip1} and WWOX and phosphorylation of p27^{Kip1} at Thr187 in late-passage *Wwox*$^{+/-}$ and *Wwox*$^{-/-}$ MEFs were determined by western blotting. β-actin was used as a loading control.

Supplementary Figure 7

a

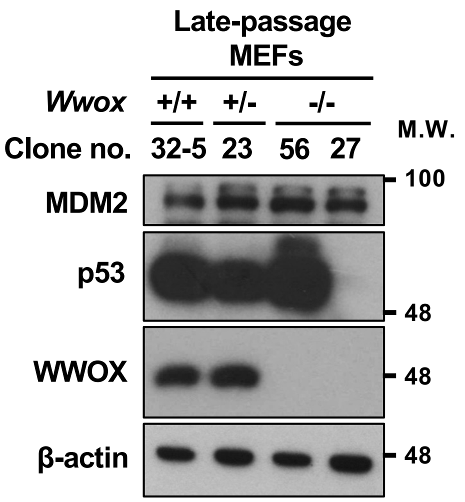

b

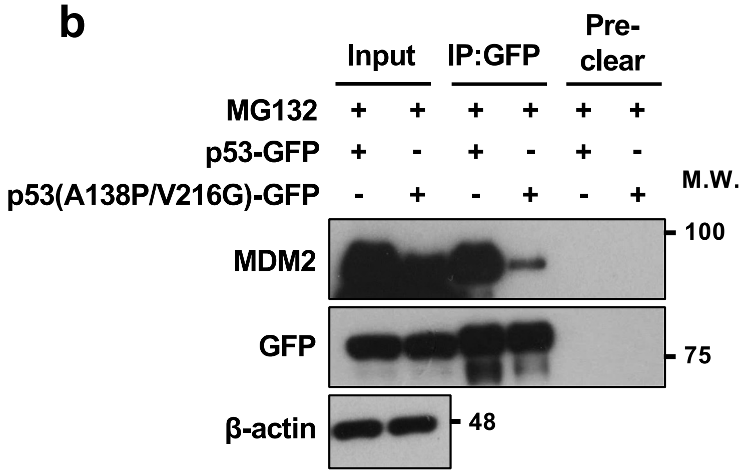

Fig. S7 MDM2 protein levels are comparable in late-passage MEFs and A138P/V216G mutations in p53 abrogate its binding to MDM2. **a** The protein expression levels of MDM2, p53 and WWOX in late-passage *Wwox*$^{+/+}$, *Wwox*$^{+/-}$ and *Wwox*$^{-/-}$ MEFs were determined by western blotting. β-actin was used as an internal control. **b** HEK293T cells transfected with a vector expressing GFP-tagged human wild-type or A138P/V216G mutant p53 were treated with MG132 (5 μM) for 14 h. The cell lysates were used for co-immunoprecipitation with an anti-GFP antibody. The presence of MDM2 in the immunoprecipitates was determined by western blotting and β-actin was used as an internal control.

Supplementary Figure 8

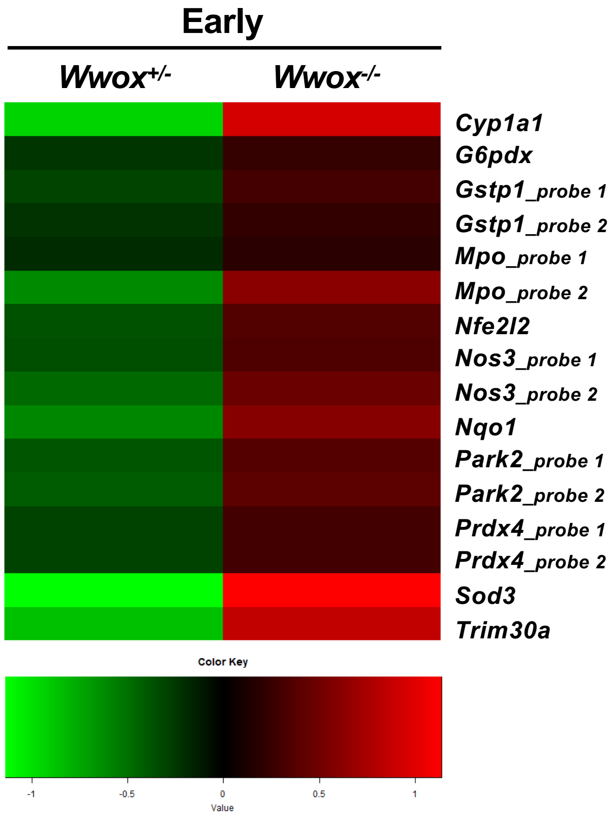

Fig. S8 The expression profile of genes associated with redox regulation in early-passage *Wwox*$^{+/-}$ and *Wwox*$^{-/-}$ MEFs. The mRNA samples from the newly isolated *Wwox*$^{+/-}$ and *Wwox*$^{-/-}$ MEFs were collected for detecting the gene expression profile using an Agilent microarray platform. The microarray ProcessedSignal data were analyzed utilizing a quantile normalization procedure and an Agilent Feature Extraction image analysis software. The heatmap results of microarray analysis are shown and the scale represents Log2 fold change of gene expression.

Supplementary Figure 9

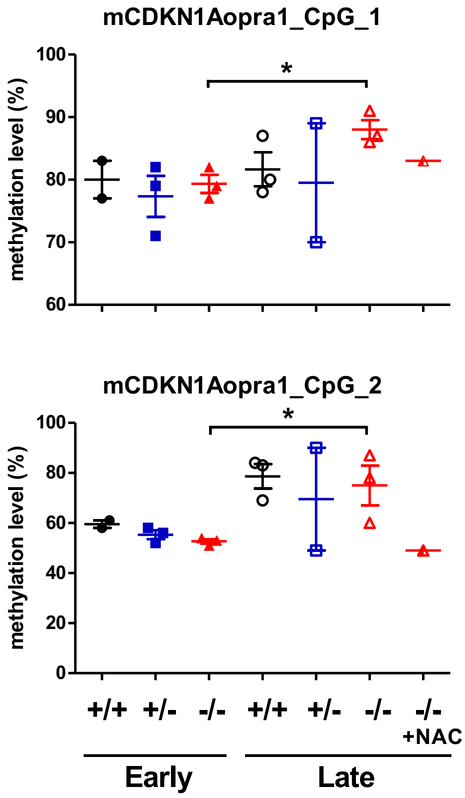

Fig. S9 The status of *p21*$^{Cip1/Waf1}$ promoter methylation in MEFs. DNA methylation of *p21*$^{Cip1/Waf1}$ promoter at specific CpG islands in *Wwox*$^{+/+}$, *Wwox*$^{+/-}$, *Wwox*$^{-/-}$ and 1 mM NAC-treated *Wwox*$^{-/-}$ MEFs at early- and late-passages was analyzed. *, P ≤ 0.05; Two-tailed *t* test.
